# Unprecedented visible light-initiated topochemical [2 + 2] cycloaddition in a functionalized bimane dye

**DOI:** 10.3762/bjoc.21.37

**Published:** 2025-03-05

**Authors:** Metodej Dvoracek, Brendan Twamley, Mathias O Senge, Mikhail A Filatov

**Affiliations:** 1 School of Chemical and Biopharmaceutical Sciences, Technological University Dublin, City Campus, Grangegorman, D07 ADY7 Dublin, Ireland; 2 School of Chemistry, Trinity College Dublin, The University of Dublin, Dublin 2, Ireland; 3 School of Chemistry, Trinity College Dublin, The University of Dublin, Trinity Biomedical Sciences Institute, 152-160 Pearse Street, Dublin D02 R590, Ireland

**Keywords:** bimane, [2 + 2] cycloaddition, fluorescence, topochemical polymerization, X-ray crystallography

## Abstract

Bimanes, a class of molecules based on the 1*H*,7*H*-pyrazolo[1,2-*a*]pyrazole-1,7-dione scaffold, were first introduced by E. M. Kosower in 1978. In this study, we report the topochemical cycloaddition of diethyl 2,6-dichloro-1,7-dioxo-1*H*,7*H*-pyrazolo[1,2-a]pyrazole-3,5-dicarboxylate (**Cl_2_B**), initiated by visible light. Crystal structure analysis confirmed that the reactive double bonds are parallel and coplanar, in line with the Schmidt criteria for topochemical cycloaddition. Additionally, two other bimane derivatives with different substitution patterns were synthesized and investigated. Our findings suggest that functionalizing bimanes to redshift their absorption maxima into the visible-light spectrum provides a promising strategy for synthesizing substituted cyclobutanes without the need for ultraviolet irradiation.

## Introduction

Topochemical polymerizations refer to polymerization reactions occurring in the solid state, which are highly dependent on molecular packing. The concept of topochemical reactions was first introduced by Kohlschütter in 1919 [1], and topochemical polymerizations were systematically studied by Schmidt and co-workers in the 1960s and 1970s [2–4]. Schmidt's pioneering work primarily focused on [2 + 2] photocycloaddition in the solid state, making it one of the earliest known examples of topochemical polymerization and a valuable method for synthesizing substituted cyclobutanes [5]. These solid-state reactions are highly influenced by the arrangement of monomers within the crystal lattice and are often reversible, requiring either high-energy light or heat for initiation [6–8]. Notably, topochemical polymerizations align with the principles of green chemistry, as they are solvent-free and do not involve toxic reagents [9].

The highly ordered and uniform nature of crystalline compounds significantly enhances the stereoselectivity and yield of topochemical reactions compared to those conducted in solution [10]. A key requirement for these reactions is the intermolecular distance between the two reactive double bonds. Schmidt’s rule, which governs topochemical [2 + 2] cycloaddition reactions, states that for the reaction to occur, the distance between the centers of the double bonds must be less than 4.2 Å, and the double bonds must be coplanar and parallel [11].

Designing topochemically active compounds is a challenging task, as the crystal packing mode must first be optimized, which can only be predicted computationally – a process that becomes increasingly complex with more intricate structures [12]. Since these reactions depend on both crystal packing and specific intermolecular distances, another key challenge lies in designing novel structures capable of undergoing topochemical reactions. Currently, topochemically active monomers are limited to large, planar aromatic molecules, aryl-substituted olefins, and polyenes, often complexed with metals. These compounds are frequently employed in the construction of metal-organic frameworks [13–15]. The limited range of compounds used thus far is likely due to reliance on their ability to undergo π-stacking interactions, which facilitate face-to-face packing. Therefore, the discovery of a new type of structure capable of undergoing this reaction, as reported here, represents a significant advancement, enabling the development of a novel scaffold for topochemically active monomers.

### Bimanes

**Figure 1:**
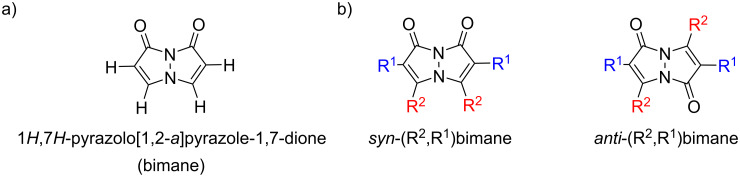
Structures of a) the unfunctionalized bimane scaffold and b) the two isomers of bimanes with their respective naming conventions.

Bimanes, a class of bicyclic compounds first synthesized by Kosower and Pazhenchevsky in 1978 [16], are based on a 1*H*,7*H*-pyrazolo[1,2-*a*]pyrazole-1,7-dione scaffold. The general structure of a bimane is depicted in Figure 1a.

**Figure 2:**
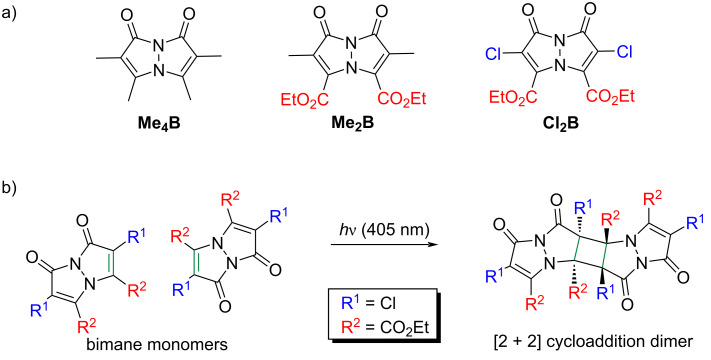
a) Structures of the bimanes studied and b) the reaction scheme of the [2 + 2] photocycloaddition of **Cl_2_B**.

The term "bimanes" is derived from the Latin ‘bi’ (two) and ‘manus’ (hand). *syn*-Bimanes are often characterized by high fluorescence quantum yields, ranging from 60% to 100% [17], while *anti*-isomers typically exhibit phosphorescence [18]. Figure 1b illustrates the two isomers along with their simplified naming conventions. Although these dyes have been known since 1978, their photochemistry has only been briefly explored. In this work, during the synthesis of a series of these dyes (Figure 2a), we observed an unprecedented intermolecular [2 + 2] cycloaddition of *syn*-(ethoxycarbonyl,chloro)bimane (**Cl_2_B**), seen in Figure 2b.

We hypothesized that this reaction occurred in the solid state, and further investigations confirmed this. To validate our findings, we employed single-crystal X-ray diffraction, which revealed the packing mode in the crystal and identified any photoproducts formed. Two additional compounds, *syn*-(ethoxycarbonyl,methyl)bimane (**Me_2_B**) and *syn*-(methyl,methyl)bimane (**Me_4_B**), were also examined for this phenomenon but did not undergo the reaction.

## Results and Discussion

### Synthesis

**Figure 3:**
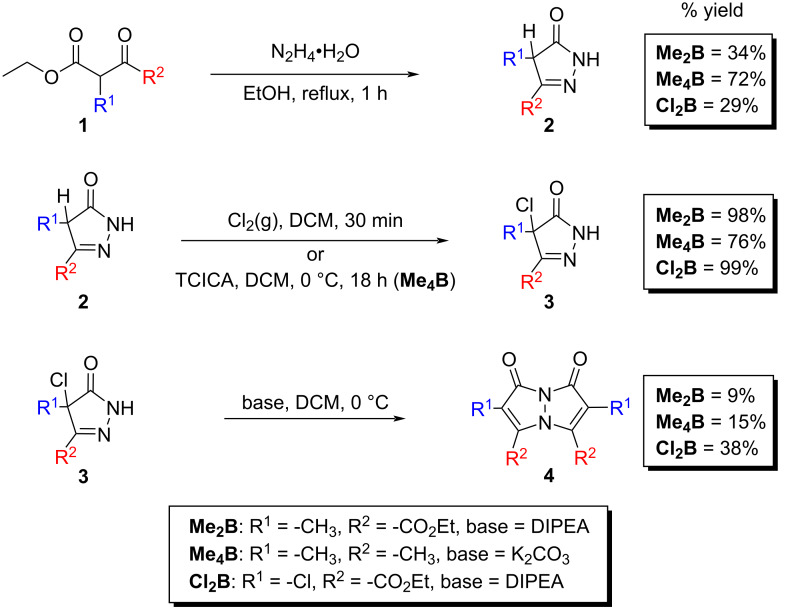
Synthetic approach to bimanes.

Bimanes are typically synthesized through a three-step process, as outlined in Figure 3. First, a β-keto ester **1** reacts with hydrazine hydrate and is heated to reflux, forming a pyrazolinone **2**. This product is then chlorinated at the α-carbon, introducing a leaving group, to yield **3**. The parent pyrazolinone **3** is subsequently treated with a base (either K_2_CO_3_ or *N,N*-diisopropylethylamine) to form the bimane **4**.

For the chlorination reactions on the synthetic path to **Cl_2_B** and **Me_2_B**, chlorine gas was generated by reacting MnO₂ with HCl and then passed through a stirred suspension of the pyrazolinone. In the case of **Me_4_B**, trichloroisocyanuric acid (TCICA) was used as the chlorinating agent as a safer alternative to chlorine gas, with the reaction conducted by stirring the pyrazolinone with TCICA in DCM for 18 hours.

Bimanes can be purified easily using column chromatography with silica gel as the stationary phase, with the compounds **Cl_2_B**, **Me_2_B**, and **Me_4_B** only requiring neat DCM as the eluent. After chromatographic purification, the ^1^H NMR of **Cl_2_B** showed 6% of an impurity, possibly the *anti*-isomer. Further purification of **Cl_2_B** reduced the impurity content to less than 3%.

### Topochemical photocycloaddition

Upon examining the crystal structure of our first sample of **Cl_2_B**, **Cl_2_B** (**A**), we observed that the compound had undergone a [2 + 2] cycloaddition reaction, with approximately 20% yield of the dimer in the crystal.

**Figure 4:**
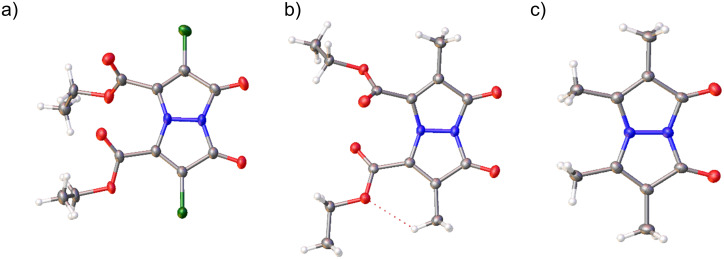
View of the molecular structures in the crystal of the functionalized bimanes studied: a) **Cl_2_B (B)**, representing 90% of the disordered asymmetric unit, b) **Me_2_B**, not disordered with dotted line representing an intramolecular hydrogen bond, and c) **Me_4_B** showing the majority occupied (55%) N–N moiety.

This was particularly notable, given that the compound had spent minimal time in solution with limited exposure to visible light, and no exposure to ultraviolet light. To further explore this phenomenon, we included two additional bimane samples, **Me_2_B** and **Me_4_B**. These samples were crystallized from a MeOH–DCM 1:1 (v/v) mixture which was left to slowly evaporate over the course of several days at room temperature, followed by single-crystal X-ray diffraction to determine their structure (Figure 4) and crystal packing mode. Extra precautions were taken to shield the compounds from light during synthesis, purification, and storage.

After crystallizing each bimane, a vial containing multiple crystals was selected for irradiation studies. **Cl_2_B (B)**, which showed 5% presence of the [2 + 2] dimer after crystallography, was irradiated, resulting in **Cl_2_B** (**C**), which showed approximately 18% dimer formation, while no dimerization was observed in the **Me_2_B** and **Me_4_B** (**C**) samples (see experimental section for details). The initial presence of the [2 + 2] dimer in the **Cl_2_B (A)** sample can be attributed to improper shielding from light, and unavoidable light exposure during crystal selection, and the X-ray measurement itself. As an additional test, the compounds were prepared as NMR samples in DMSO-*d*_6_ and irradiated with 405 nm LED. Irradiation in the solution phase did not result in any dimerization; the respective ¹H NMR spectra remained unchanged compared to the neat compounds (Figures S1, S3, and S5, Supporting Information File 1), confirming that the reaction occurs selectively in the solid state. **Cl_2_B** was also irradiated in DCM with the same 405 nm LEDs, in a quartz fluorescence cuvette. A UV–vis spectrum was then taken at time intervals of 15–30 minutes for a total of 3 h. The UV–vis monitoring revealed that the absorbance decreases as irradiation continues, indicating photodegradation; however, no evidence of a [2 + 2] cycloaddition product was seen in the spectra (Supporting Information File 1, Figure S15). Following this, more ^1^H NMR irradiation studies were conducted, where each bimane was dissolved in dichloromethane-*d*_2_, and irradiated using the same 405 nm light source for 1.25 h. The NMR spectra showed that no [2 + 2] dimer yield was achieved (Supporting Information File 1, Figures S8–S10). Further details on this are available in the experimental section.

This suggests that the reaction is topochemical, with the bimane’s reactivity dependent on its crystal packing, since no [2 + 2] cycloaddition product was seen in the spectrum. While the reaction may also depend on the reactivities of the different bimane molecules, discussing the crystal packing may bring insights into identifying other structures which may undergo this reaction also.

**Figure 5:**
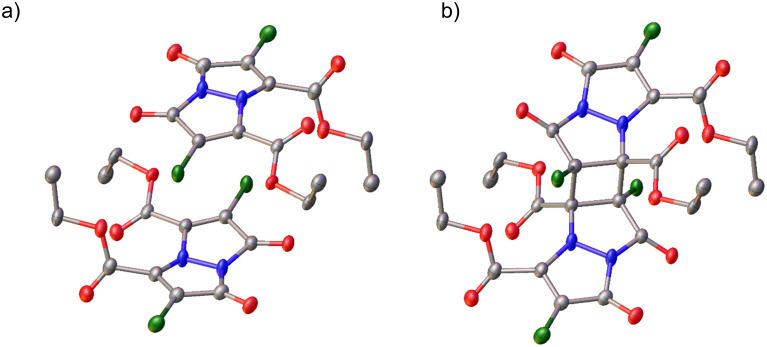
View of the molecular structure in the crystal of a) symmetry generated by inversion bimanes **Cl_2_B (B)**. Each bimane is only 90% occupied and b) **Cl_2_B (B)** topochemical [2 + 2] cycloaddition product, symmetry generated also by inversion. This moiety is 5% occupied in total. Hydrogen atoms omitted for clarity. Both disordered moieties were modelled using bond length restraints and displacement parameter restraints.

It should be noted that all three **Cl_2_B** structures are, by necessity, modelled as highly disordered, and the whole molecule is disordered for **Cl_2_B (A)**. Crystallographic bond length restraints and constraints were used in each model and bond lengths and angle accuracy are affected by the disorder model (see Supporting Information File 1 for further details). In the case of **Cl_2_B** (all crystals, Figure 5), the torsion angle between the two double bonds in the bimane is 0(0°), indicating that the reacting double bonds are coplanar and parallel. Furthermore, the intermolecular distances between the reacting carbons are identical. The distance between two carbon double bonds in **Cl_2_B** (**B**) was determined to be 3.487(4) Å (Table 1).

The key question is why did **Cl_2_B** react and **Me_2_B** did not. One possible explanation lies in the light source used for irradiation. The emission wavelength (405 nm) is within 2 nm of the λ_max_ of **Cl_2_B** (as a solution in DCM), allowing it to absorb the initiation light with much greater intensity. Additionally, **Cl_2_B** has a higher molar extinction coefficient (6100 M^−1^ cm^−1^) compared to **Me_2_B** (4700 M^−1^ cm^−1^) at their λ_max_ values of 403 nm and 394 nm in DCM, respectively [19]. This difference in light absorption is further exacerbated by the fact that the irradiation wavelength is not aligned with the λ_max_ of **Me_2_B**. Calculating from the reported value, the extinction coefficient of **Me_2_B** at 405 nm is approximately 4500 M^−1^ cm^−1^. As a result, initiating the reaction in **Me_2_B** is not as efficient, and may require a shorter wavelength of higher intensity. However, as reported by Kosower et al. [18], bimanes are prone to photoreactions under UV light irradiation, undergoing rearrangement and photodegradation, so there may be a risk of degradation if irradiation using UV light is tried. Alternatively, the lack of a [2 + 2] cycloaddition of **Me_2_B**, may be due to its photophysical properties not allowing for a reaction to occur, rather than the irradiation conditions. Inefficient intersystem crossing (or complete lack thereof) and short singlet-state lifetimes can both affect the efficiency of a photoreaction [20].

### Crystal packing

**Figure 6:**
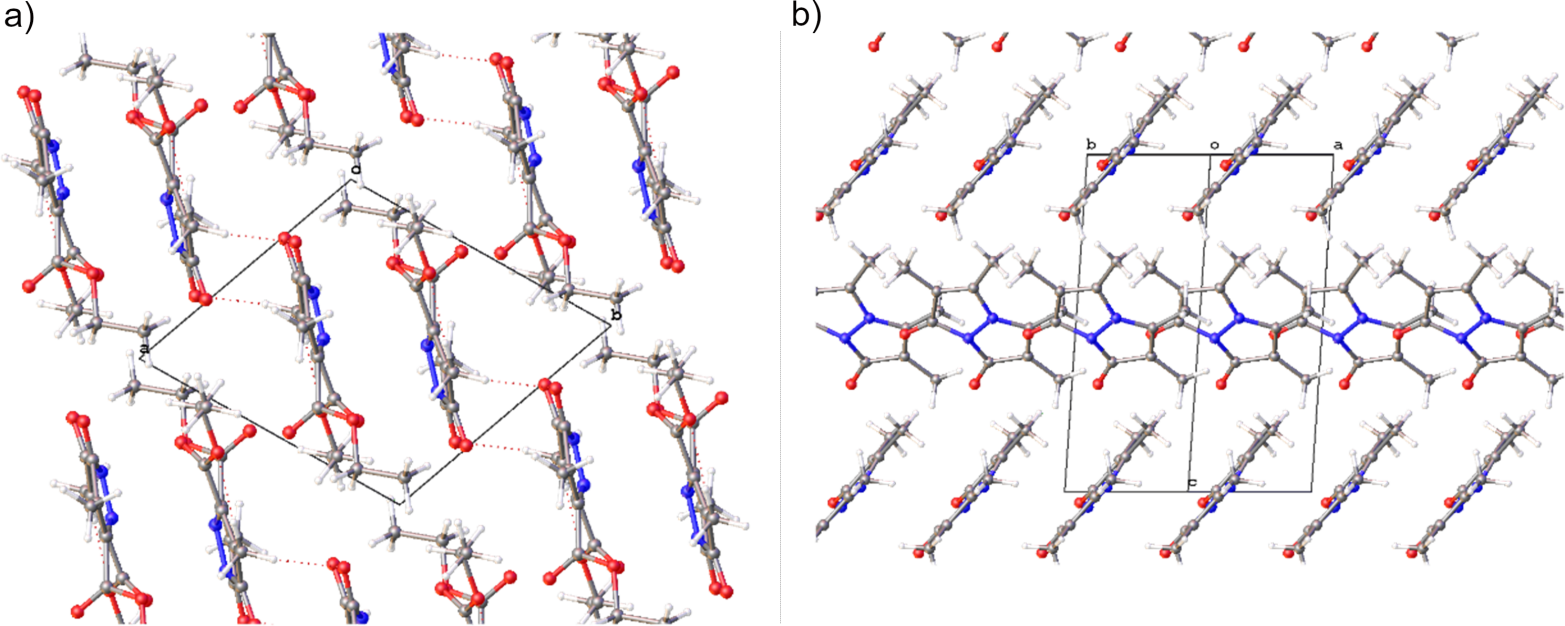
View of the packing of the unit cells of a) **Me_2_B** viewed normal to the *c*-axis and b) **Me_4_B** viewed normal to (110). Only the majority occupied moiety of the N–N bridge is shown in **Me_4_B**.

Similar to **Cl_2_B**, the potentially reactive double bonds in **Me_2_B** are coplanar and parallel, with a torsion angle of 0(0°) (crystallographic unit cell can be seen in Figure 6a). However, in **Me_2_B**, the bonds are further apart, with an intermolecular separation of 3.862(2) Å. Although this is still within the range for a potential reaction, no reaction was observed. In contrast, **Me_4_B** lacks the proper alignment of the reactive double bonds and is therefore not expected to undergo this topochemical reaction (Figure 6b). Examination of the reported structure of 9,10-dioxa-*syn*(carboethoxy,methyl)bimane in the CCDC database (BESGAH) [19,21], shows that it crystallizes in the monoclinic (*P*2(1)/*c* space group, unlike **Me_2_B** which crystallizes in *P*-1 and the molecules pack in a different arrangement, where the double bonds are neither coplanar nor parallel. This product was obtained from propan-2-ol, which may have influenced the crystallization kinetics. The reactive packing mode of **Cl_2_B** is enhanced by the hydrogen bonding where the ester groups play a role by increasing the number of hydrogen bonds (C–H···O, 3.18(5)–3.62(6) Å) The intermolecular distances between the C–H groups and the chlorine atoms in **Cl_2_B (A)** are C13A–H13···Cl1A, 3.69(5), and C18A–H18C···Cl2A, 3.69(3) Å, indicating further molecular attraction [22].

A previously reported bimane, *syn*-(H,Cl)bimane (BIYGUL), reveals a ribbon-like packing pattern, which arises from a hydrogen-bonding network [23], which also features a coplanar and parallel arrangement of the potentially reactive double bonds. Due to these characteristics, the reaction site can be represented by a parallelogram (Table 1), allowing for a more detailed comparison of the bond alignment.

**Table 1:** Parallelogram representation data of the reactive double bonds in a) all **Cl_2_B** structures, **Me_2_B**, and also *syn*-(H,Cl)bimane.

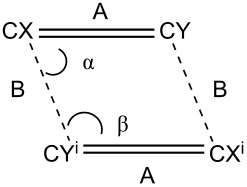	X	Y	A	B	α	β	% bimane

**Cl** **_2_** **B(A)**	C6a	C7a	1.346(7)	3.556(8)	78.5(3)	101.4(3)	58
**Cl** **_2_** **B(B)**	C3a	C4a	1.354(4)	3.487(4)	77.5(2)	102.5(2)	90
**Cl** **_2_** **B(C)**	C6a	C7a	1.350(8)	3.489(14)	77.9(4)	102.1(4)	63
**Me** **_2_** **B**	C6	C7	1.3592(18)	3.8622(18)	63.92(8)	116.08(8)	100
***syn*** **-(H,Cl)bimane**	C2	C3	1.345	3.635	73.0	106.9	100

As shown in Table 1, while the crystallographic center between the bonds meets the Schmidt criteria, the reactive carbons in **Cl_2_B** are better aligned compared to the two unreactive bimanes. The packing arrangement in **Me_2_B** and **Me_4_B** is shown in Figure 6, indicating the alignment of the bimanes in the solid state.

This reactive packing arrangement is also observed in an *anti*-bimane previously synthesized by Blenderman et al. In *anti*-(Me,Br)bimane (FOSREK), coplanar and parallel double bonds are present; however, the distance between these bonds is outside the acceptable range (4.2 Å) [24]. This finding, along with the re-examination of several previously synthesized bimane compounds, suggests that substituting the bimane chromophore with groups that sufficiently redshift the absorption maximum could result in a wide range of compounds capable of undergoing topochemical [2 + 2] cycloaddition reactions initiated by visible light.

A number of related bimane structures have been reported. These include *anti*-(Ph,Cl)bimane (CPDZBO10) where the Ph residues impart a bowl-shaped bimane distortion and *syn*-(Ph,Cl)bimane [25], *anti*-(H,Me)bimane (DMOCDO10) and *syn*-(Me,Me)bimane (DXABIM10) [26], a room temperature structure of **Me_4_B** (TNZBCO10) [27], and a planar *syn*-(H,ethynyl)bimane (WAYHEJ) [28].

### Optical properties

The optical properties of the three bimanes were measured to investigate differences in their excited state properties, as shown in Table 2. These properties help to explain the variation in photoreactivity. Quantum yields were measured using **Me_2_B** as an internal reference, with a value of 33% in acetonitrile, as initially determined by Kosower [19]. While tentative, the low fluorescence quantum yields of these compounds may suggest potential triplet-state formation, which is involved in the proposed mechanism for the cycloaddition reaction. Although **Me4B** could theoretically form triplet states, given its fluorescence quantum yield of 51.7–67.3%, its crystallographic structure is not conducive to this reaction, regardless of its photophysical properties.

**Table 2:** Steady-state spectroscopic data from **Me_4_B**, **Me_2_B**, and **Cl_2_B** recorded in acetonitrile (ACN), dichloromethane (DCM), and toluene (TOL). Fluorescence quantum yields (Φ_fl_) calculated with respect to **Me_2_B** in acetonitrile (Φ_fl_ = 33%) [19]. Shoulders (sh) on emission spectrum are indicated in parentheses.

	solvent	λ_abs_ (nm)	λ_em_ (nm)	Φ_fl_

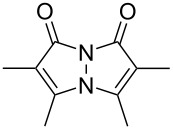 **Me** **_4_** **B**	TOL	365	417	53.4%
	DCM	369	428	67.3%
	ACN	368	433	51.7%
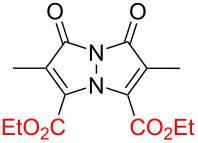 **Me** **_2_** **B**	TOL	398	462 (492 sh)	49.5%
	DCM	394	462 (502 sh)	63.4%
	ACN	391	463 (495 sh)	33.0%
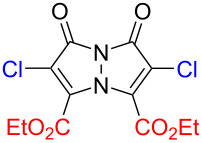 **Cl** **_2_** **B**	TOL	414	474 (502 sh)	40.4%
	DCM	404	473 (505 sh)	47.6%
	ACN	401	476 (505 sh)	20.2%

**Figure 7:**
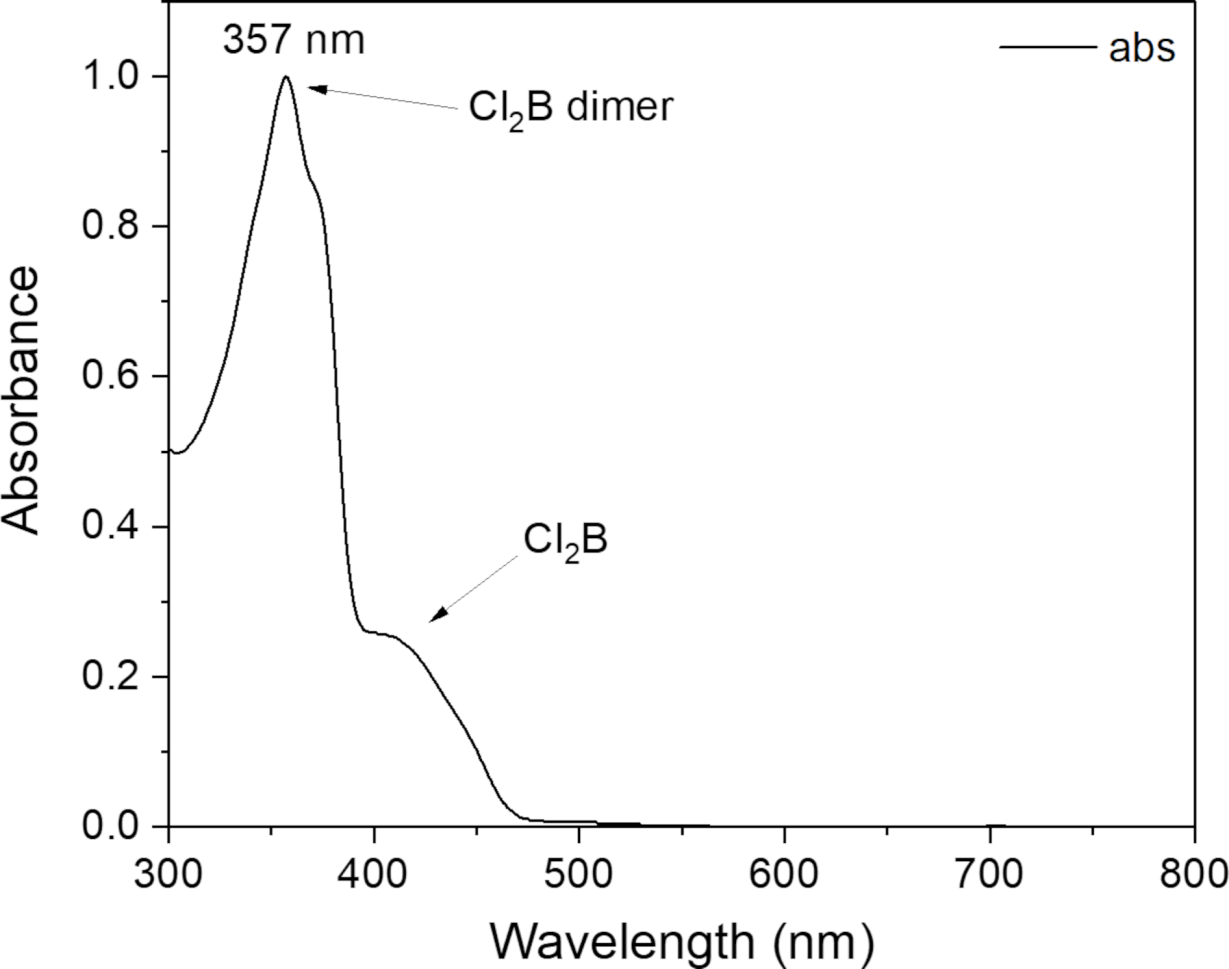
UV–vis spectrum of **Cl_2_B** after irradiation in DCM.

While a pure sample of the dimer has not yet been isolated, a UV–vis spectrum of **Cl_2_B** after 1.75 hours of irradiation in DCM was recorded. The peak corresponding to the remaining monomer was observed, along with a new peak blueshifted by 47 nm compared to the parent bimane (Figure 7). The hypsochromic shift in this reaction means that provided correct irradiation wavelength is used, a 100% yield of the dimer could theoretically be achieved.

### Tentative mechanism

**Figure 8:**
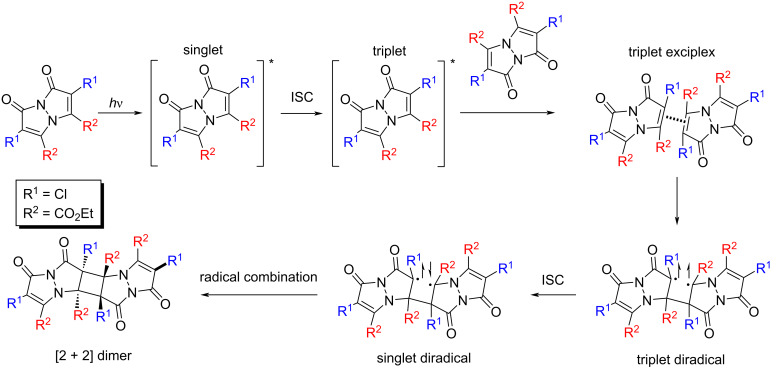
Proposed mechanism for the topochemical [2 + 2] photocycloaddition of **Cl_2_B**.

The observed reaction likely follows a five-step mechanism, as suggested by previous studies [29–30]. The proposed pathway begins with the excitation of a bimane molecule to its singlet-excited state, followed by intersystem crossing (ISC) to the triplet state. The triplet state then forms an excimer complex with a neighboring bimane molecule, leading to the formation of a single bond and generating a triplet diradical. This triplet diradical undergoes another ISC, returning to the singlet state, which then forms a second single bond (Figure 8). This mechanism, involving both singlet-excited states and triplet states via ISC, is consistent with the relatively low fluorescence quantum yields observed for **Cl_2_B**.

## Conclusion

Following the observation of the topochemical cycloaddition of **Cl_2_B**, two other bimane dyes, **Me_2_B** and **Me_4_B**, were synthesized and investigated for their ability to undergo this reaction when initiated by visible light. A notable feature of this reaction is that it occurs only in the solid state, not in solution. Although **Me_2_B** had the correct alignment of double bonds, meeting the Schmidt criteria, the reaction did not proceed under 405 nm excitation. This may be due to steric effects, a lack of molecular attraction, or an insufficient molar extinction coefficient at that wavelength. **Me_4_B** did not have the correct packing mode for a topochemical [2 + 2] cycloaddition to occur, and as such no reaction was observed. Synthesizing bimanes with functional groups that redshift the absorption maximum into the visible-light spectrum could yield a variety of structures capable of undergoing this reaction.

## Experimental

### Synthesis

The studied bimanes were synthesized following the approach reported by Kosower and co-workers [17,19], and an alternative chlorination method developed by Neogi et al. [31]. The synthetic details can be found in Supporting Information File 1. Compounds were crystallized from a MeOH–DCM 1:1 (v/v) solution which was left to evaporate at room temperature over the course of a few days.

### Photophysical properties

Fluorescence spectra were measured on a Horiba FluoroMax Plus spectrofluorimeter. UV–vis spectra were measured on a Shimadzu UV-1900i UV–vis spectrophotometer. Absorption and fluorescence spectra are presented in Supporting Information File 1 (Figures S12–S14).

For the fluorescence quantum yield measurements, solutions of each bimane were prepared, and the concentration was adjusted so that the absorbance at the right shoulder of the band at 360 nm falls within the range of 0.03–0.08. A fluorescence spectrum was then recorded, exciting at 370 nm, using 1 nm excitation and emission slits. The emission was recorded in the range of 370–850 nm. This was done for each solvent: DCM, acetonitrile, and toluene. The quantum yields were calculated by using **Me_2_B** in acetonitrile as the reference (Φ_fl_ = 33%) [19].

Quantum yields were calculated using the following formula (Equation 1):


[1] $$\Phi_{\text{s}}=\Phi_{\text{ref}}\times \frac{{\text{Abs}}_{\text{ref}}}{{\text{Abs}}_{\text{s}}}\times \frac{{\text{Area}}_{\text{s}}}{{\text{Area}}_{\text{ref}}}\times {\left(\frac{\eta_{\text{s}}}{\eta_{\text{ref}}}\right)}^{2}$$

where Φ_s_ is the fluorescence quantum yield of the analyte and Φ_ref_ is the fluorescence quantum yield of the reference, Abs is the absorbance at the excitation wavelength, Area is the area under the fluorescence spectrum and η is the refractive index of the solvent.

### Single crystal X-ray diffraction studies

Crystals of **Me_4_B**, **Cl_2_B** (**A**), (**B**), (**C**), and **Me_2_B** were mounted on a MiTeGen micromount with NVH immersion oil. Data were collected from a shock-cooled single crystal at 100(2) K on a Bruker Apex Kappa Duo Imus Cu Kα Kappa diffractometer with a microfocus sealed X-ray tube using mirror optics as a monochromator and an APEX2 detector. The diffractometer was equipped with an Oxford Cobra low temperature device and used Cu Kα radiation (λ = 1.54178 Å). All data were integrated with SAINT and a multi-scan absorption correction using SADABS was applied [32–33]. Structures were solved by dual methods using SHELXT and refined by full-matrix least-squares methods against *F*^2^ by SHELXL using Olex2 [34–35]. All non-hydrogen atoms were refined with anisotropic displacement parameters. All hydrogen atoms were refined isotropic on calculated positions using a riding model with their *U*_iso_ values constrained to 1.5 times the *U*_eq_ of their pivot atoms for terminal sp^3^ carbon atoms and 1.2 times for all other carbon atoms. Disordered moieties were refined using bond length restraints and displacement parameter restraints [21]. Full crystal and refinement details are given in Supporting Information File 1, section 4).

### Irradiation tests

The bimanes were crystallized from a MeOH–DCM 1:1 (v/v) solution and were allowed to evaporate at room temperature over the course of several days. In a typical experiment, the crystals were then irradiated for variable periods of time using 405 nm LED (75 mW cm^−2^) in an Elegoo Mercury Plus Wash and Cure (turntable chamber). Samples were placed approximately 8 cm far from the light source. The average light power measured at this distance was 5 mW cm^−2^. During the irradiation, the crystals changed their appearance from light yellow to dark orange within 15 minutes.

An alternative method for solid-state irradiation involved dissolving 5–10 mg of the bimane in a minimal amount of acetone, which was then evaporated in a 25 mL round-bottomed flask, forming a seemingly amorphous solid spread across the flask. The entire flask was then irradiated using 405 nm LEDs in the turntable chamber.

### Solution-phase irradiation tests

For solution-phase irradiations, 10 mg of each compound was dissolved in DMSO-*d*_6_ and placed into NMR tubes. The samples were irradiated directly inside the tubes, using the same light source as for the solid-state samples.

Another series of tests involved dissolving 5–15 mg of each bimane in dichloromethane-*d*_2_ in an NMR tube. The tubes were irradiated for 1.25 h using the same light source as for the solid-state samples.

**Cl_2_B** irradiation was also monitored using UV–vis spectroscopy, where the concentration was adjusted such that the absorbance is 2.164 at the λ_max_. The solution was added into a quartz fluorescence cuvette, and irradiated for 15 minutes at a time using the same 405 nm LEDs in a turntable chamber.

## Supporting Information

File 1 Spectroscopic and crystallographic information as well as synthetic details.

## Data Availability

All data that supports the findings of this study is available in the published article and/or the supporting information of this article.
