# Supplementary material for: Unprecedented visible light-initiated topochemical [2 + 2] cycloaddition in a functionalized bimane dye
Source: Beilstein J Org Chem. 2025 Mar 5;21:500–9. doi: 10.3762/bjoc.21.37 (PMC11897657; doi:10.3762/bjoc.21.37)
Supplement: File 1 — Spectroscopic and crystallographic information as well as synthetic details. [file Beilstein_J_Org_Chem-21-500-s001.pdf]

BEILSTEIN JOURNAL OF ORGANIC CHEMISTRY

Supporting Information

for

Unprecedented visible light-initiated topochemical [2 + 2] cycloaddition in a functionalized bimane dye

Metodej Dvoracek, Brendan Twamley, Mathias O. Senge and Mikhail A. Filatov

Beilstein J. Org. Chem. **2025,** *21,* 500–509. doi:10.3762/bjoc.21.37

Spectroscopic and crystallographic information as well as synthetic details

License and Terms: This is a supporting information file under the terms of the Creative Commons Attribution License (https://creativecommons.org/licenses/by/4.0). Please note that the reuse, redistribution and reproduction in particular requires that the author(s) and source are credited and that individual graphics may be subject to special legal provisions.
The license is subject to the *Beilstein Journal of Organic Chemistry* terms and conditions: (https://www.beilstein-journals.org/bjoc/terms)

Table of contents

1. General procedures S1
   1.1. Synthesis S1
      1.1.1. Diethyl 2,6-dimethyl-1,7-dioxo-1*H*,7*H*-pyrazolo[1,2-*a*]pyrazole-3,5-dicarboxylate (Me_2B). S1
      1.1.2. Diethyl 2,6-dichloro-1,7-dioxo-1*H*,7*H*-pyrazolo[1,2-*a*]pyrazole-3,5-dicarboxylate (Cl_2B) ... S2
      1.1.3. 2,3,5,6-Tetramethyl-1*H*,7*H*-pyrazolo[1,2-*a*]pyrazole-1,7-dione (Me_4B) S3
2. NMR spectra S5
3. Mass spectra S12
4. Absorption and emission spectra S13
   4.1. Dichloromethane S13
   4.2. Acetonitrile S13
   4.3. Toluene S13
   4.4. UV–vis monitoring of Cl_2B irradiation S14
5. X-ray crystallography S15
6. References S23

1. General procedures

Fluorescence spectra were measured on a Horiba FluoroMax Plus spectrofluorimeter. UV–vis spectra were measured on a Shimadzu UV-1900i UV–vis spectrophotometer. Quartz cuvettes (path length = 1 cm) were used for both. NMR spectra were recorded on a Bruker Avance III 500 MHz spectrometer.

Compounds used for synthesis (diethyl 2-methyl-3-oxosuccinate, ethyl 2-methyl-3-oxobutanoate, diethyl oxalacetate, DIPEA, hydrazine hydrate) were purchased from Merck.

1.1. Synthesis

All compounds were synthesized using modified literature procedures [1-3]

1.1.1. Diethyl 2,6-dimethyl-1,7-dioxo-1*H*,7*H*-pyrazolo[1,2-*a*]pyrazole-3,5-dicarboxylate (Me_2B)

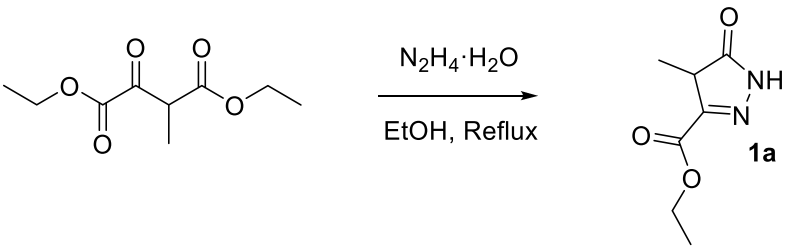

Diethyl 2-methyl-3-oxosuccinate (5 g, 0.0247 mol, 1 equiv) was dissolved in EtOH (40 mL). Hydrazine hydrate (1.235 g, 0.0247 mol, 1 equiv) in 2 mL EtOH was added dropwise to the solution. The reaction mixture was stirred at rt for 1 h, and precipitation of product was observed. The mixture was then heated at 60 °C for 0.5 h. Upon cooling, the precipitate was filtered through a glass filter and was allowed to dry at rt for 2 hours, yielding ethyl 4-methyl-5-oxo-4,5-dihydro-1*H*-pyrazole-3-carboxylate (**1a**, 1.41 g, 0.0042 mol 17% yield). The analytical data are in agreement with those previously reported in the literature [1].

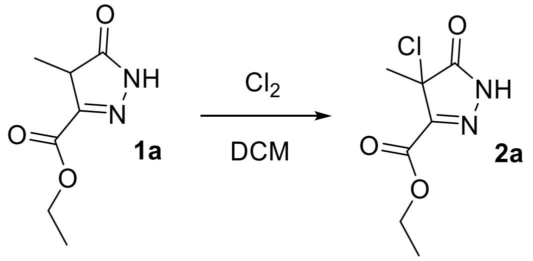

To generate chlorine gas, concentrated hydrochloric acid (80 mL) was added to a bed of solid MnO_2, slowly, dropwise via an addition funnel. The gas was first bubbled through concentrated sulfuric acid, and subsequently passed through a suspension of **1a** (2.07 g, 0.0121 mol) in ≈ 20 mL DCM. The bubbling was continued until the solid fully dissolved. The solvent was then removed under reduced pressure, yielding an oil, which was cooled to rt and solidified, resulting in a white crystalline solid of ethyl 4-chloro-4-methyl-5-oxo-4,5-dihydro-1*H*-pyrazole-3-carboxylate (**2a**, 2.45 g, 0.012 mol, 98% yield). The analytical data are in agreement with those previously reported in the literature [1].

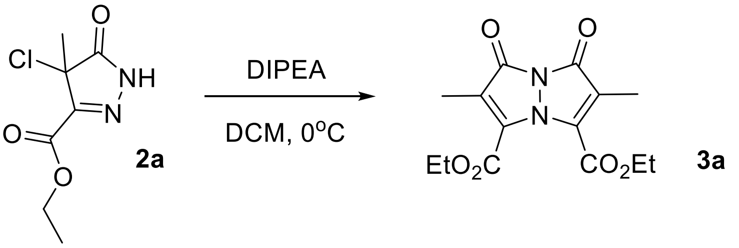

The product **2a** (1.028g, 0.005 mol, 1 equiv) was dissolved in ≈ 20 mL DCM. The atmosphere was replaced with nitrogen, and the reaction mixture was cooled to 0 °C. *N*,*N*-Diisopropylethylamine (1.008 g, 0.007 mol, 1.4 equiv) was dissolved in 5 mL DCM, which was added dropwise to the solution of **2a**. The solution turned a deep red-black color, after which the solvent was removed under reduced pressure. The crude product was loaded onto a DCM-packed silica column and chromatographed using DCM. Fractions exhibiting fluorescence were isolated and combined, and the solvent was removed under reduced pressure yielding **Me_2B** (0.208 g, 0.00020 mol, 8% yield). ^{1}H NMR (400 MHz, $CDCl_3$): δ 4.40 (q, J = 7.2 Hz, 4H), 2.06 (s, 6H), 1.40 (t, J = 7.2 Hz, 6H). ^{13}C NMR (101 MHz, $CDCl_3$) δ 159.68, 158.77, 139.03, 120.63, 63.07, 14.00, 8.10. The analytical data are in agreement with those previously reported in the literature [1].

1.1.2. Diethyl 2,6-dichloro-1,7-dioxo-1*H*,7*H*-pyrazolo[1,2-*a*]pyrazole-3,5-dicarboxylate (Cl_2B)

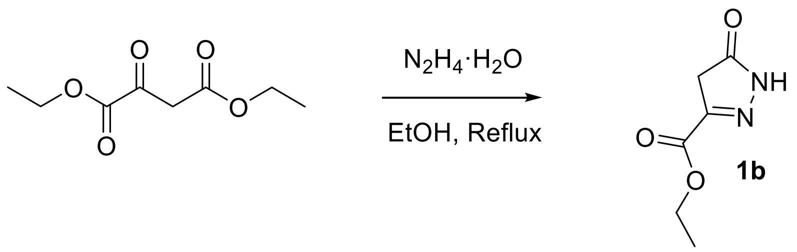

Diethyl oxalacetate (5 g, 0.0266 mol, 1 equiv) was dissolved in EtOH (40 mL). Hydrazine hydrate (1.33 g, 0.0266 mol, 1 equiv) in 2 mL EtOH was added dropwise to the solution, and the reaction mixture was subsequently heated and kept at reflux for 40 minutes. The solvent was removed under reduced pressure and the product was recrystallized from hot water to yield white crystals of ethyl 5-oxo-4,5-dihydro-1*H*-pyrazole-3-carboxylate (**1b**, 1.199 g, 0.0077 mol, 29%). The analytical data are in agreement with those previously reported in the literature [1].

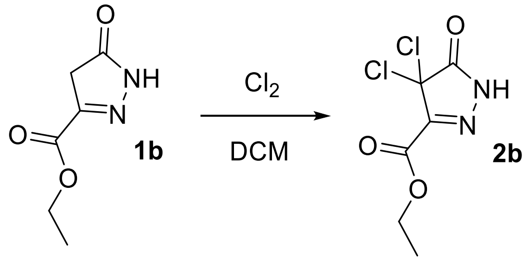

The product **1b** (0.633 g, 0.00405 mol) was added to DCM, forming a suspension. The same procedure for forming chlorine gas was used as per the procedure for **Me2B**, yielding ethyl 4,4-dichloro-5-oxo-4,5-dihydro-1*H*-pyrazole-3-carboxylate (**2b**, 0.904 g, 0.0040 mol, 99%). The analytical data are in agreement with those previously reported in the literature [1].

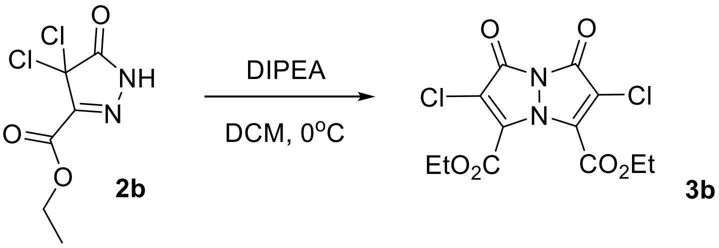

N,N-Diisopropylethylamine (682 µL, 0.00392 mol, 1 equiv) was dissolved in 10 mL DCM and added dropwise to **2b** (0.881 g, 0.00392 mol, 1 equiv) in 10 mL DCM under constant stirring. Once the solution turned red-black the reaction was stopped and solvent was removed in vacuo. The product was then loaded onto a column, which was loaded with DCM-packed silica. DCM was used as the eluent. A fluorescent yellow/green fraction was then isolated, and the solvent was removed under reduced pressure, yielding yellow crystals of *syn*-(carboethoxy,chloro)bimane (0.265 g, 0.00074 mol, 38% yield). An impurity in a concentration of 6% was observed in the initial ^{1}H NMR spectrum. A 100 mg portion of the product was purified using preparative thin layer chromatography, using DCM as the eluent, reducing the impurity to <3%. ^{1}H NMR (400 MHz, DMSO): δ 4.41 (q, *J* = 7.1 Hz, 4H), 1.31 (t, *J* = 7.1 Hz, 6H). ^{13}C NMR (101 MHz, $CDCl_3$) δ 156.51, 154.05, 138.47, 115.01, 64.16, 13.93. The analytical data are in agreement with those previously reported in the literature. **[1]**

1.1.3. 2,3,5,6-Tetramethyl-1*H*,7*H*-pyrazolo[1,2-*a*]pyrazole-1,7-dione (Me4B)

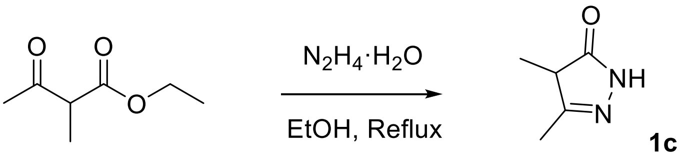

Ethyl 2-methyl-3-oxobutanoate (17.323g, 0.1202 mol, 1 equiv) was dissolved in 60 mL EtOH and heated to 50 °C. Hydrazine hydrate (8.772 g, 0.1281 mol, 1.07 equiv) was added to the solution dropwise. Upon complete addition, the reaction mixture was stirred for 1 h at 50 °C, during which time a white precipitate formed. The precipitate was then isolated via filtration in vacuo and washed with 2 mL EtOH. The solid was dried for 16 h in an oven at 30 °C, yielding 4,5-dimethyl-2,4-dihydro-3*H*-pyrazol-3-one (**3a**, 9.712 g, 0.0865 mol, 72%). The analytical data are in agreement with those previously reported in the literature [2].

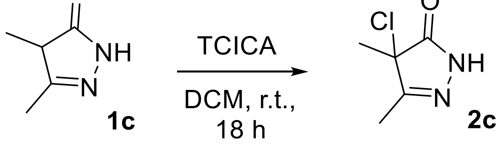

Compound **3a** (1.521 g, 0.0136 mol, 1 equiv) was dissolved in 30 mL DCM and cooled to 0 °C. Solid trichloroisocyanuric acid (TCICA, 1.889 g, 0.00512 mol, 0.38 equiv) was added incrementally over a period

of 40 min while stirring. Upon complete addition, the reaction mixture was stirred for 18 h at rt, forming a precipitate of cyanuric acid. The precipitate was removed via filtration in vacuo. Solvent was removed from the filtrate at reduced pressure, yielding 4-chloro-4,5-dimethyl-2,4-dihydro-3*H*-pyrazol-3-one (**3b**, 0.567 g, 0.0038 mol, 28%). The analytical data are in agreement with those previously reported in the literature [3].

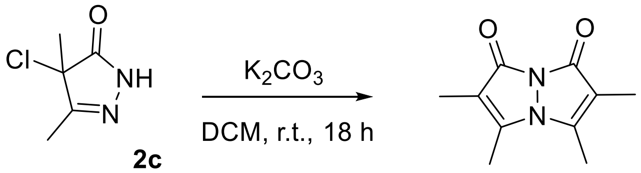

2c

3c

Compound **3b** (0.4607 g, 0.00314 mol, 1 equiv) was dissolved in 20 mL DCM and the solution cooled to 0 °C. K_2CO_3 (3.447 g, 0.025 mol, 8 equiv) was added to 20 mL DCM, forming a suspension. It was added to the solution of **3b** dropwise over the course of 1 h. The reaction mixture was then allowed to reach room temperature and left to stir for 72 h. Remaining K_2CO_3 was removed by in vacuo filtration, and solvent was removed from the filtrate under reduced pressure. The crude product was purified on a column, using DCM-packed silica with a DCM–EtOAc 10:1 mixture as the eluent. The isolated fractions were combined and solvent was removed under reduced pressure, yielding a yellow solid composed of **Me₄B** (0.092 g, 0.00047 mol, 30%). ^{1}H NMR (400 MHz, $CDCl_3$): δ 2.30 (s, 6H), 1.84 (s, 6H). ^{13}C NMR (101 MHz, $CDCl_3$) δ 160.85, 145.85, 112.62, 11.90, 6.80. The analytical data are in agreement with those previously reported in the literature [2].

2. NMR spectra

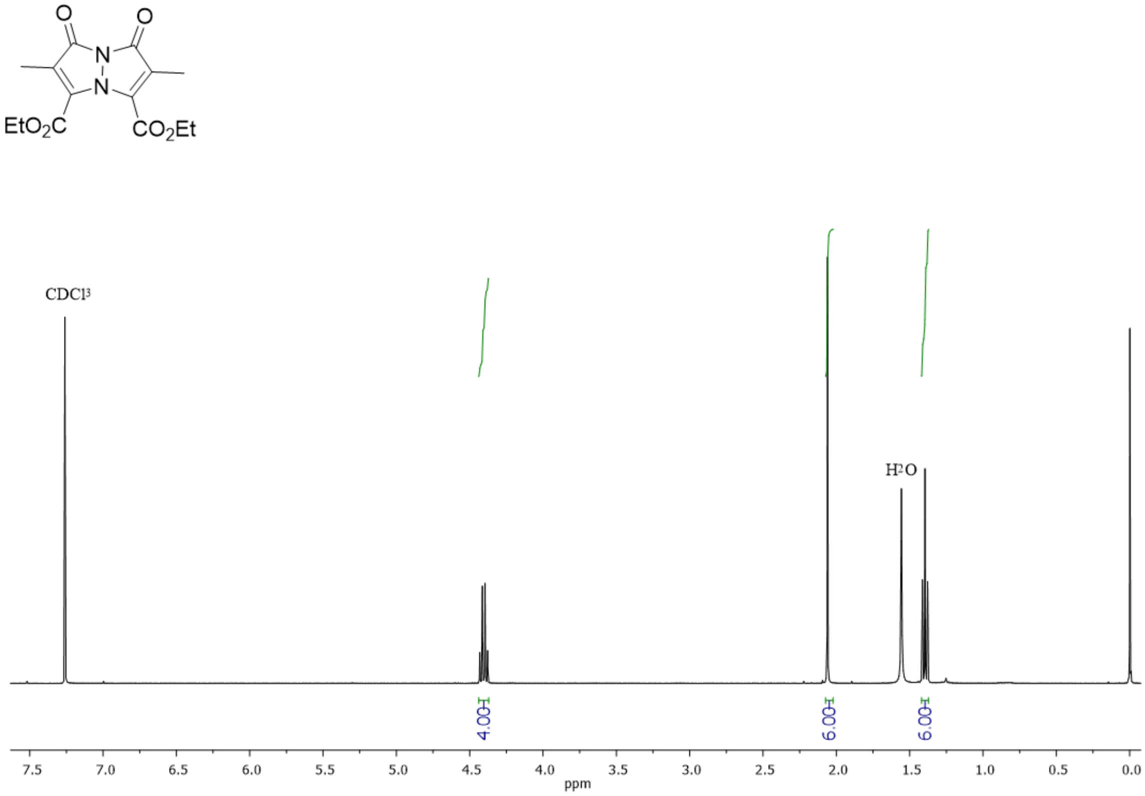

Figure S1. 400 MHz ^{1}H NMR of **Me2B** in $CDCl_3$.

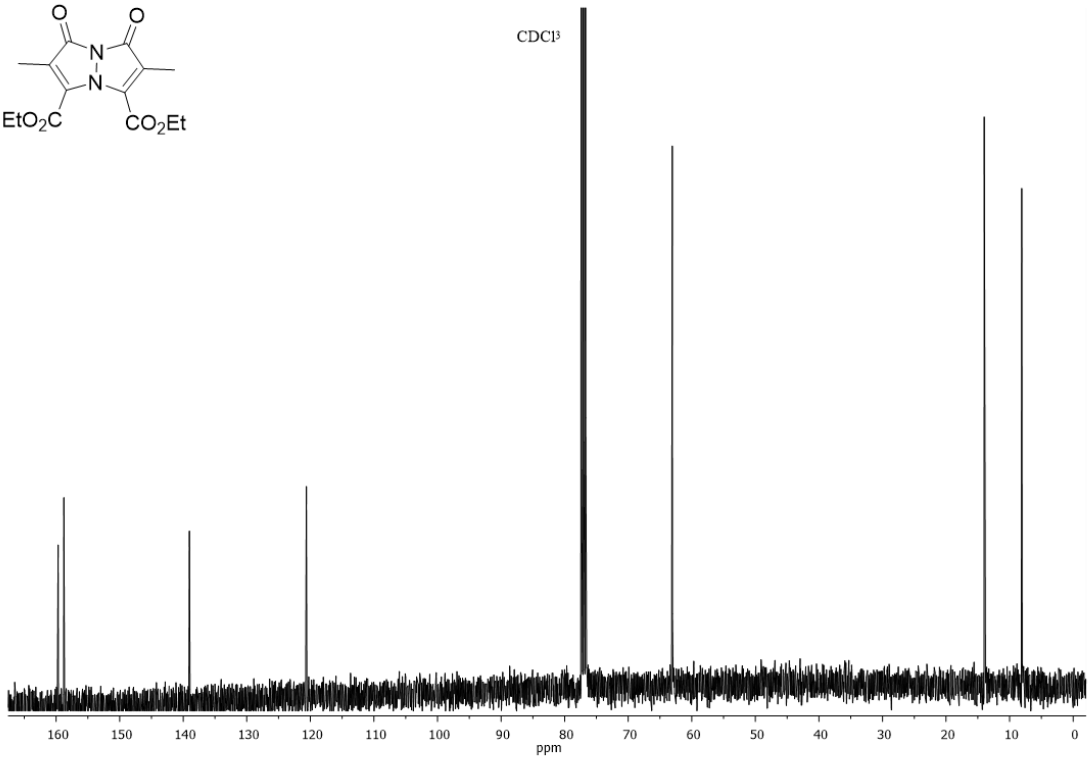

Figure S2. 101 MHz ^{13}C NMR of **Me2B** in $CDCl_3$.

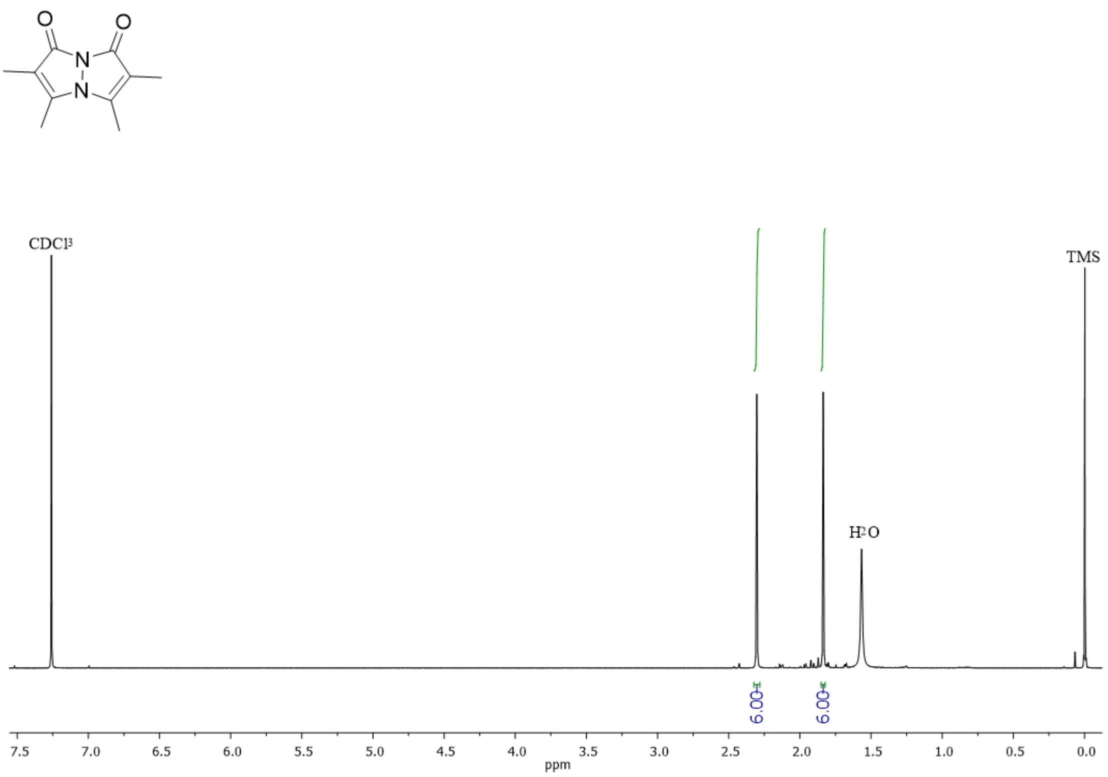

Figure S3. 400 MHz ^{1}H NMR of **Me$_4$B** in $CDCl_3$.

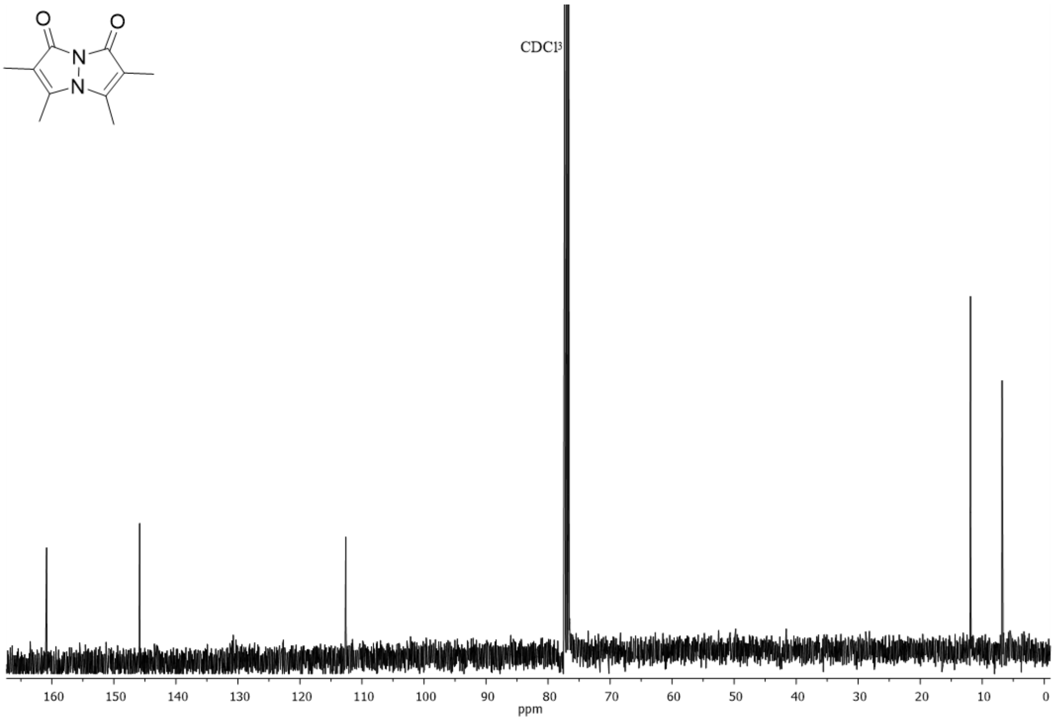

Figure S4. 101 MHz ^{13}C NMR of **Me$_2$B** in $CDCl_3$.

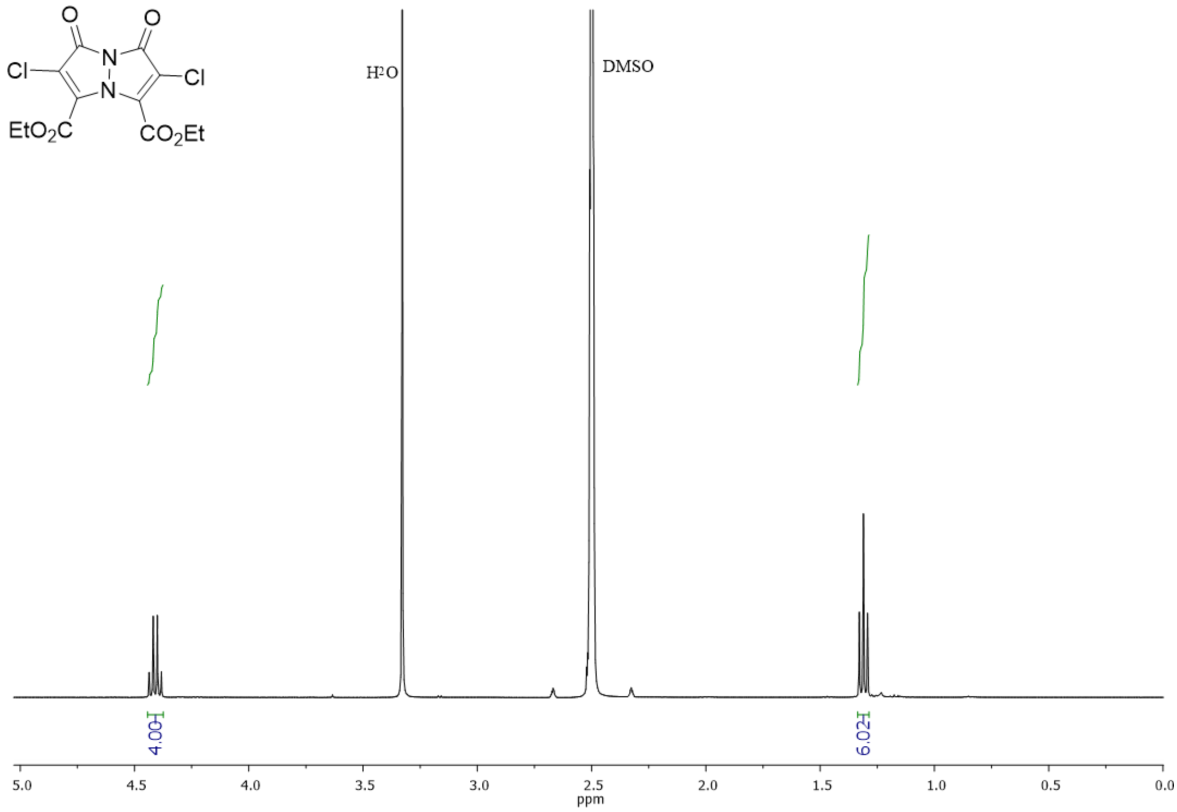

Figure S5. 400 MHz ^{1}H NMR of **Cl$_2$B** in d^6-DMSO after crystallization from MeOH – DCM.

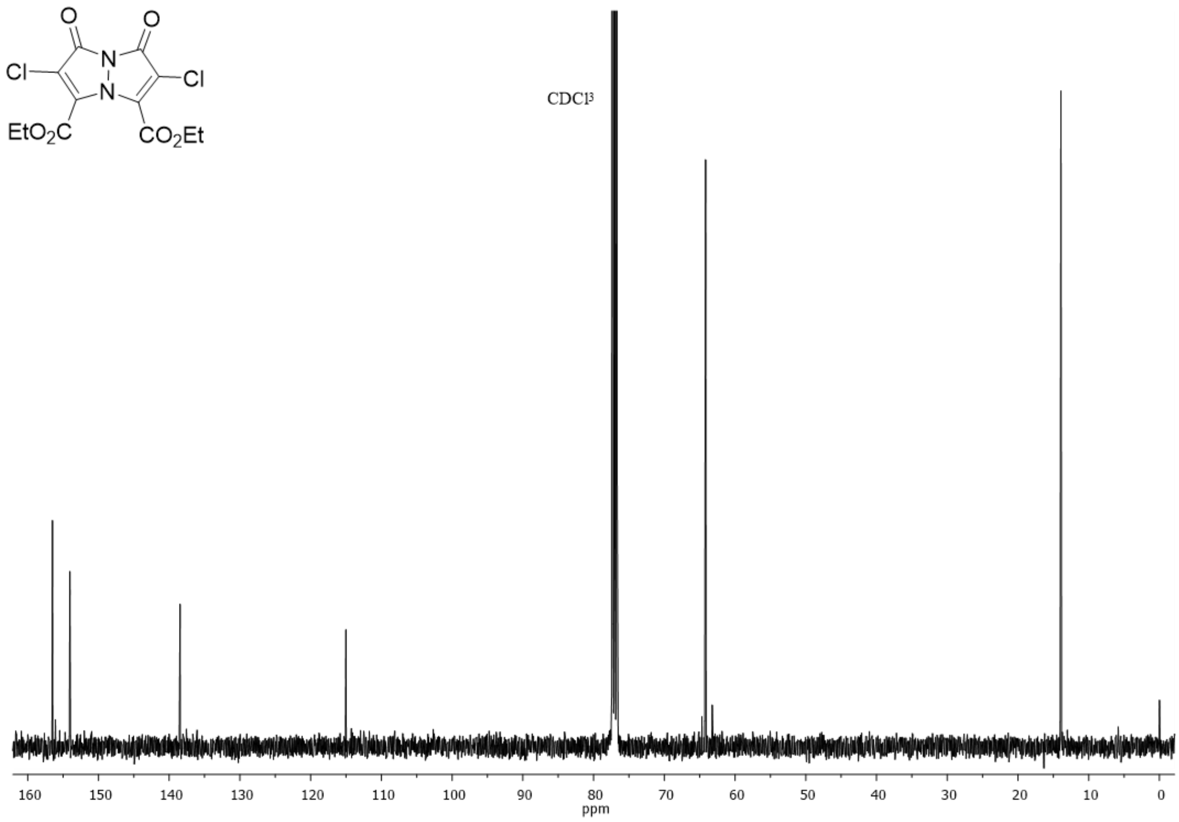

Figure S6. 101 MHz ^{13}C NMR of **Cl$_2$B** in $CDCl_3$.

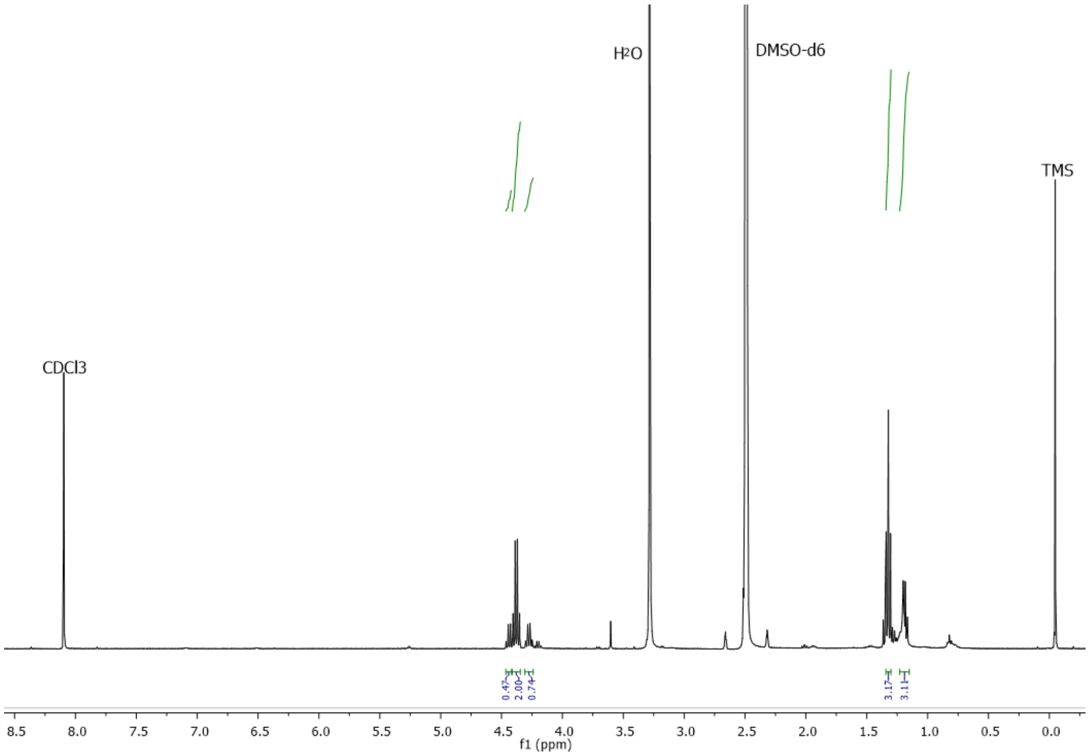

Figure S7. 400 MHz ^{1}H NMR of **Cl_2B** sample after irradiation in crystal phase for 1 h with halogen lamp. Solvent: d_6-DMSO – $CDCl_3$ mixture.

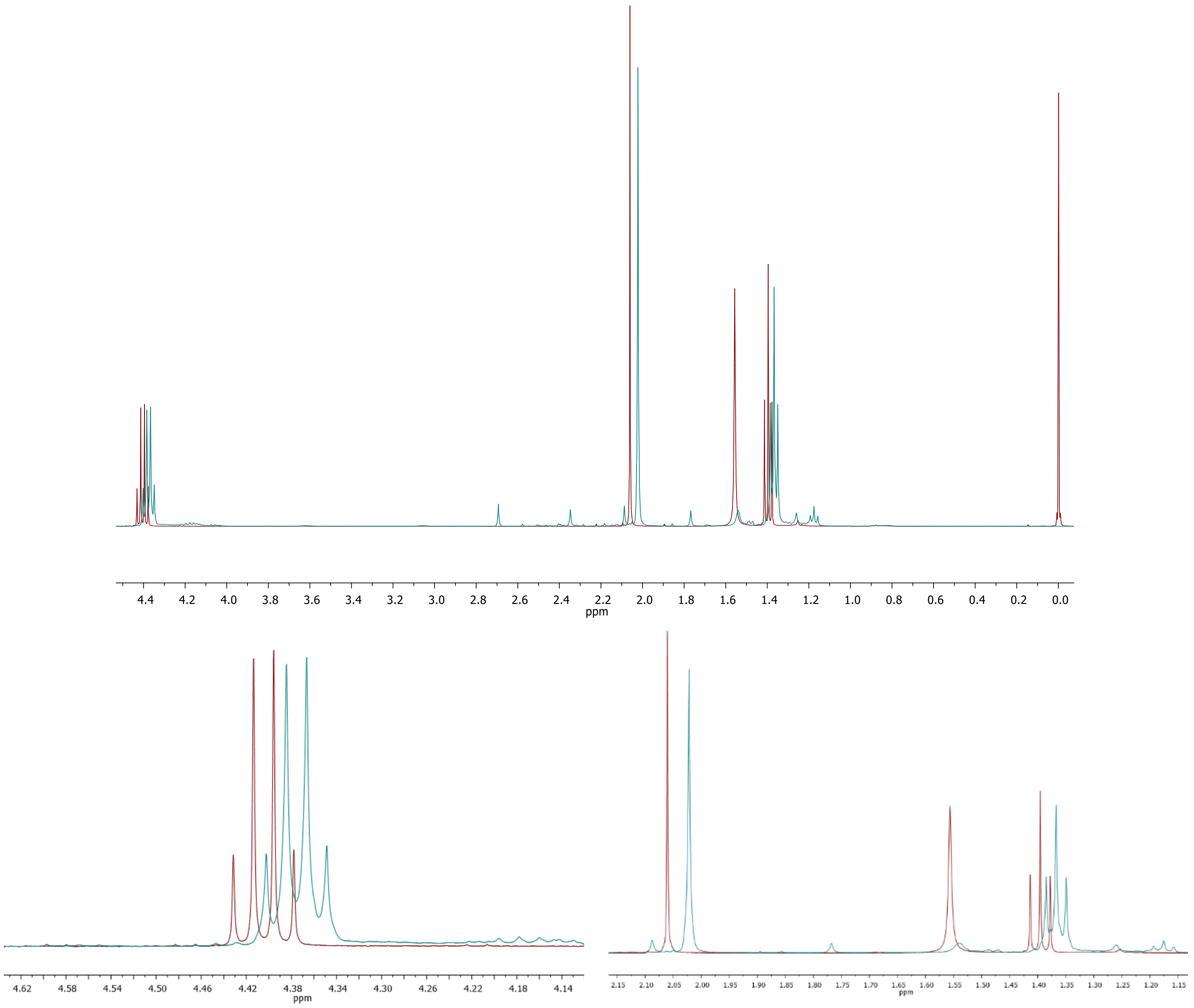

Figure S8. Overlaid 400 MHz ^{1}H NMR spectra of (red) **Me_2B** reference in $CDCl_3$ (not irradiated) and (teal) **Me_2B** solution in dichloromethane-d_2 irradiated with 405 nm LEDs for 1.25 h. Solvent peaks omitted for clarity.

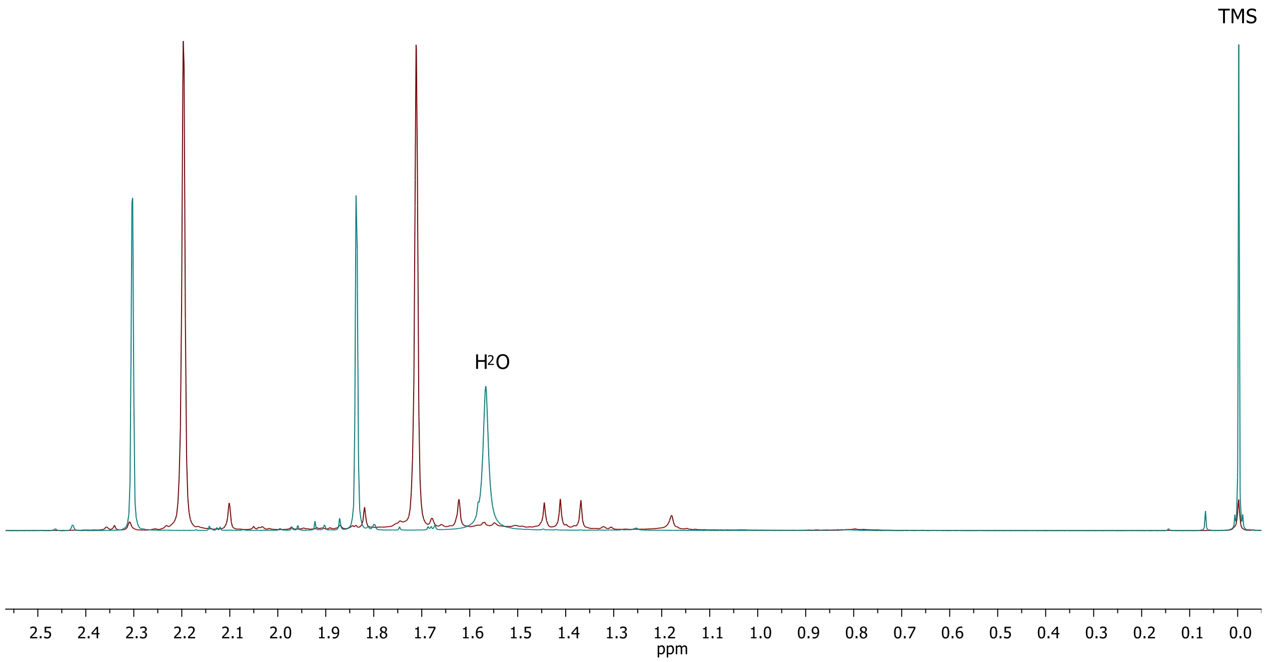

Figure S9. 400 MHz ^{1}H NMR spectra of (teal) reference **Me$_4$B** (not irradiated) in $CDCl_3$ and (red) **Me$_4$B** solution in dichloromethane-d_2 irradiated with 405 nm LEDs for 1.25 h. Solvent peaks omitted for clarity.

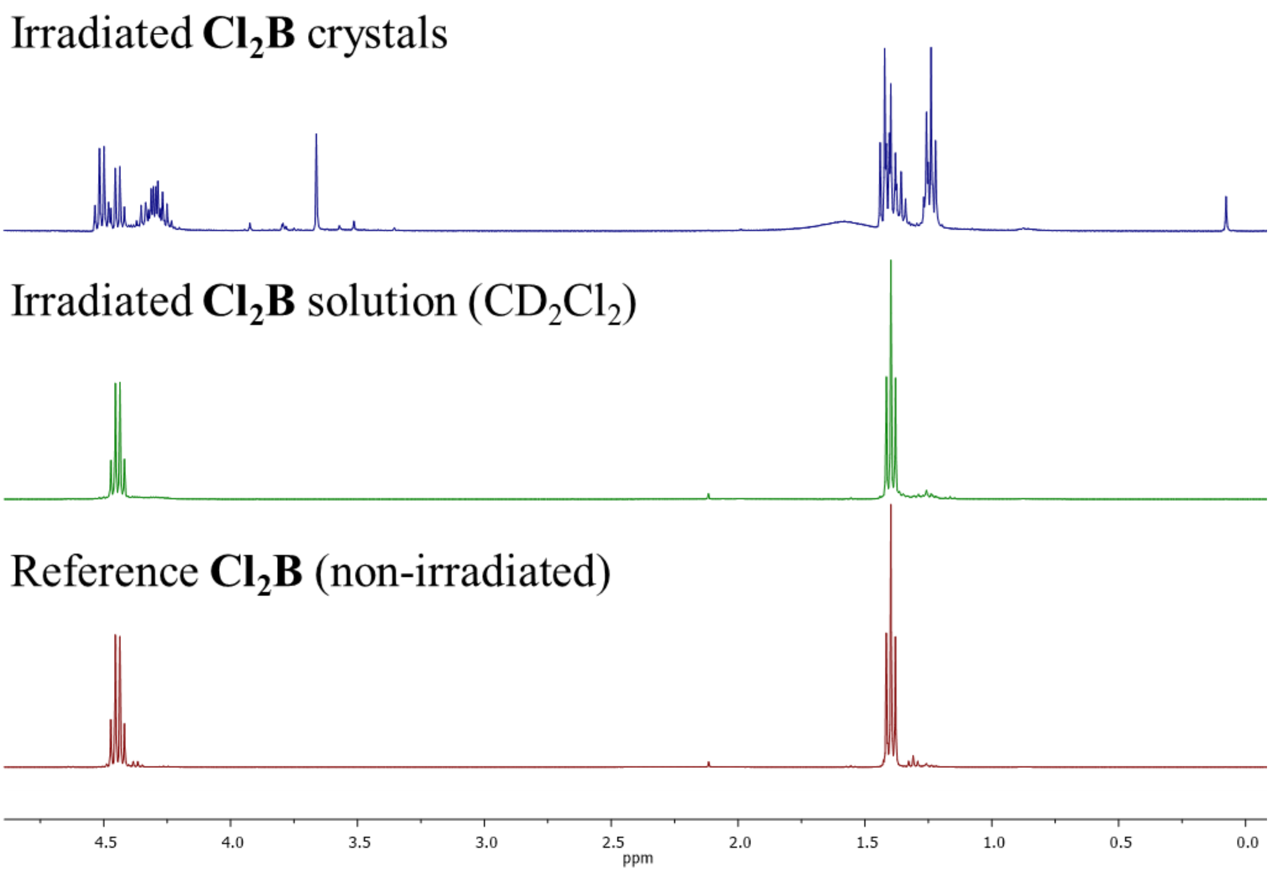

Figure S10. ^{1}H NMR spectra of (red) reference **Cl_2B** (not irradiated) in dichloromethane-*d_2*, (green) **Cl_2B** irradiated with 405 nm LEDs for 1.25 h in dichloromethane-*d_2* and (blue) **Cl_2B** crystals irradiated with 405 nm LEDs for 1.25 h (NMR spectrum in dichloromethane-*d_2*).

3. Mass spectra

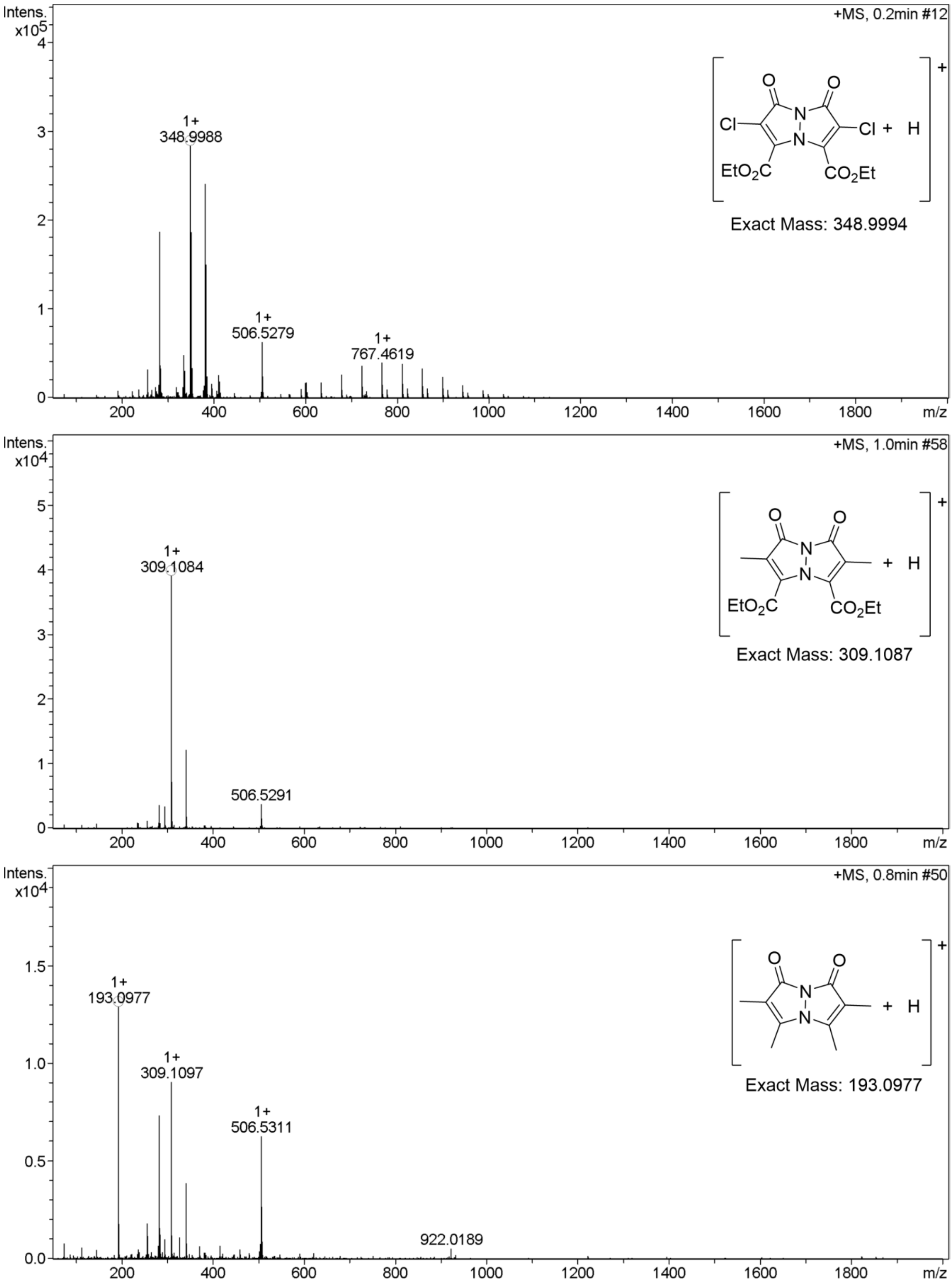

Figure S11. Mass spectra of the three reported bimanes, **Cl_2B** (top), **Me_2B** (middle) and **Me_4B** (bottom).

4. Absorption and emission spectra

4.1. Dichloromethane

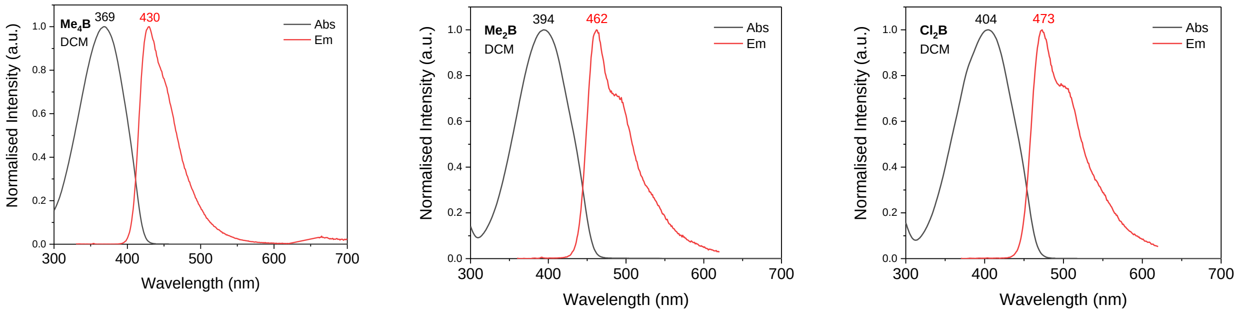

Figure S12. Absorption and emission spectra of bimanes in dichloromethane. λ_{exc} (**Me$_2$B, Cl$_2$B**) = 360 nm, λ_{exc} (**Me$_4$B**) = 330 nm.

4.2. Acetonitrile

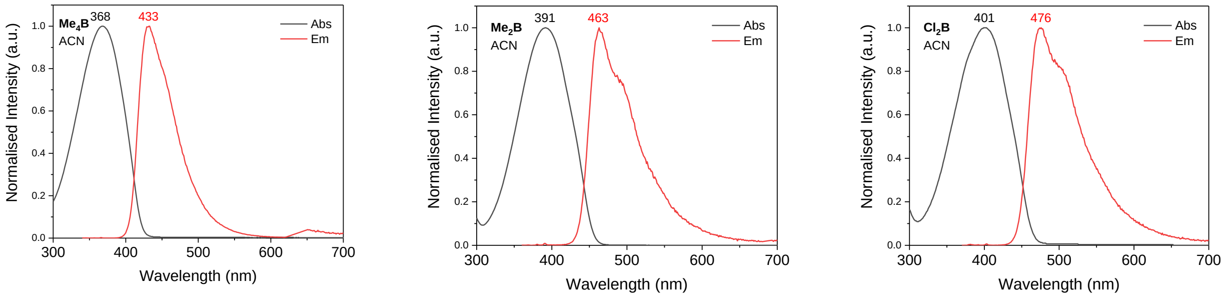

Figure S13. Absorption and emission spectra of bimanes in acetonitrile. λ_{exc} (**Me$_2$B, Cl$_2$B**) = 360 nm, λ_{exc} (**Me$_4$B**) = 330 nm.

4.3. Toluene

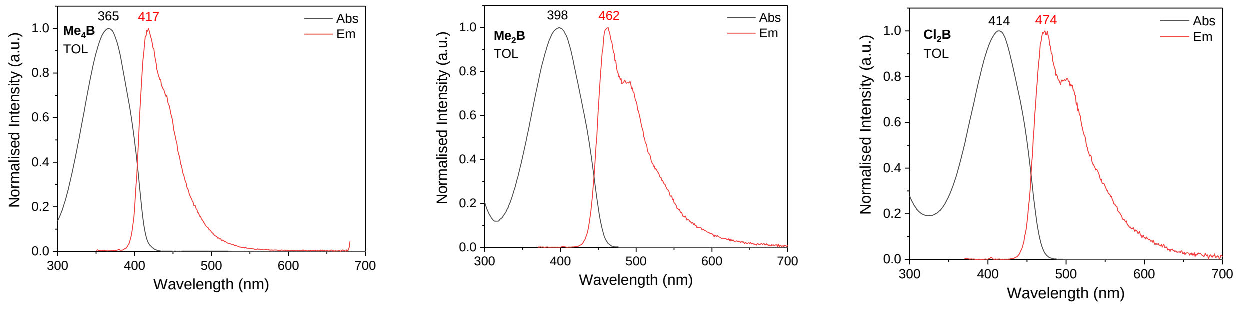

Figure S14. Absorption and emission spectra of bimanes in toluene. λ_{exc} (**Me$_2$B, Cl$_2$B**) = 360 nm, λ_{exc} (**Me$_4$B**) = 330 nm.

4.4. UV–vis monitoring of Cl_2B irradiation

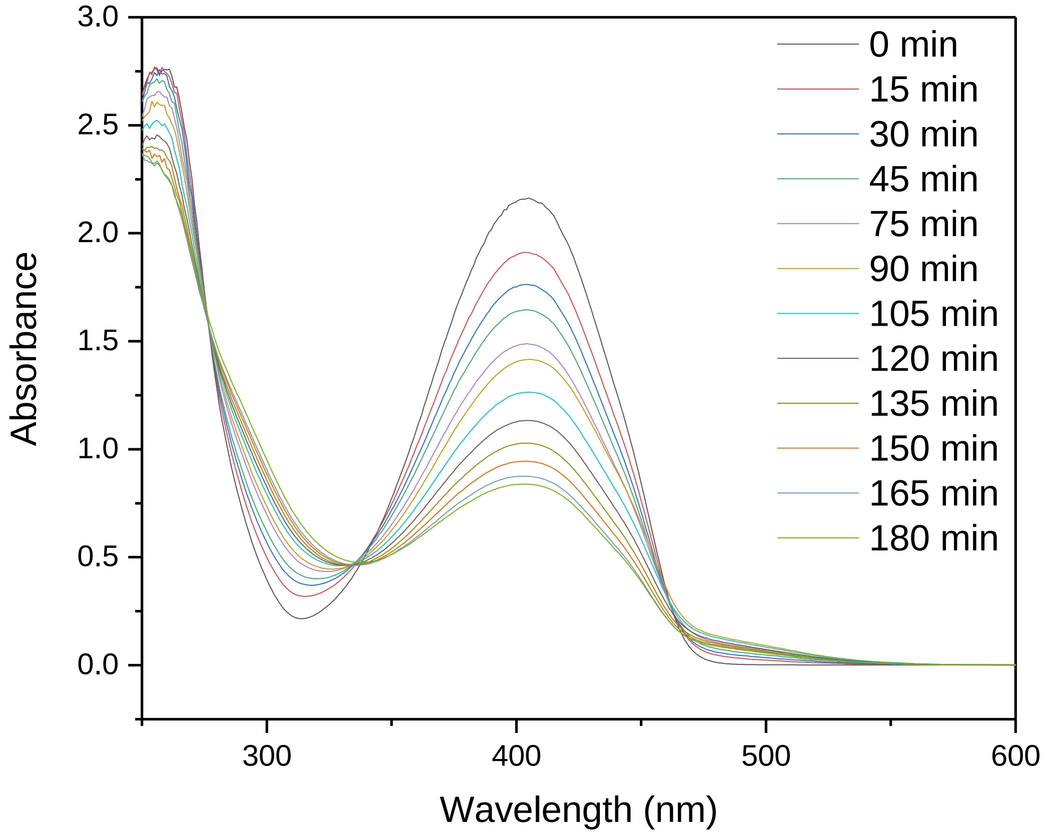

Figure S15. UV-Visible spectra of **Cl_2B** irradiated with 405 nm LEDs at time intervals in dichloromethane inside a quartz fluorescence cuvette.

5. X-ray crystallography

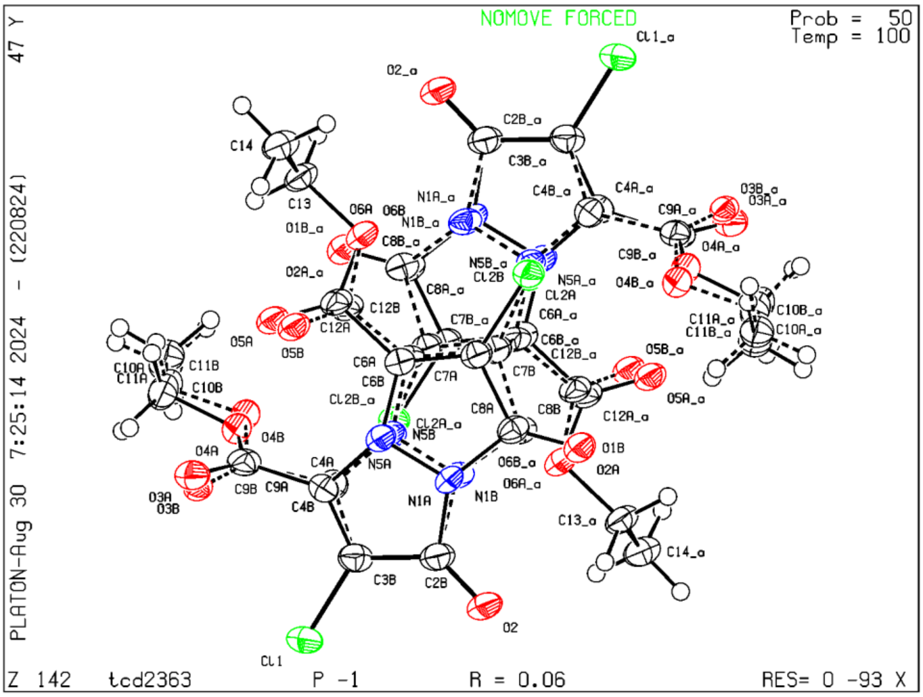

Figure S16. ORTEP plot of **Cl$_2$B (C)** with atoms labelled.

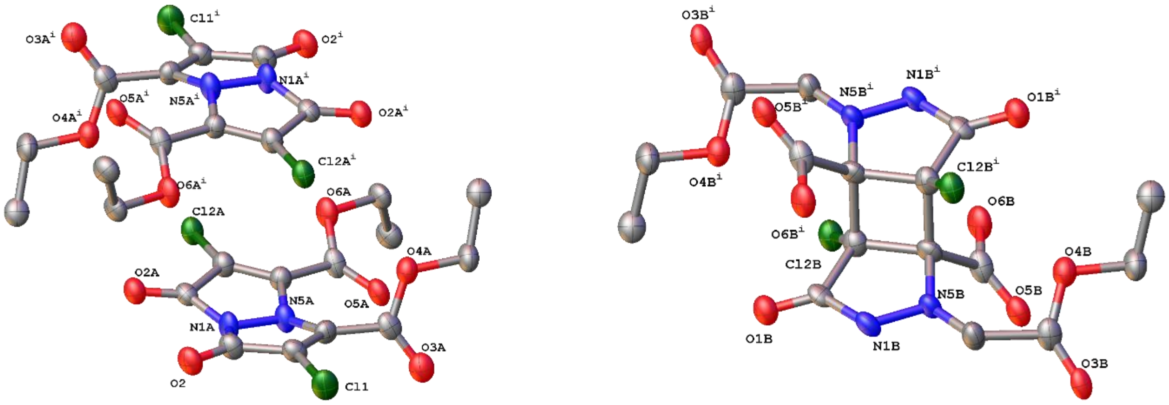

(a) (b)

Figure S17. Individual images of each disordered moiety by symmetry generation in **Cl$_2$B (C)** with (a) majority occupied moiety bimane, 63%, second bimane generated by symmetry and (b) symmetry generated minority occupied moiety cycloaddition product, 18.5 %. Symmetry transformation i = −X, 1−Y, 1−Z. Heteroatoms labelled only. Displacement shown at 50% probability.

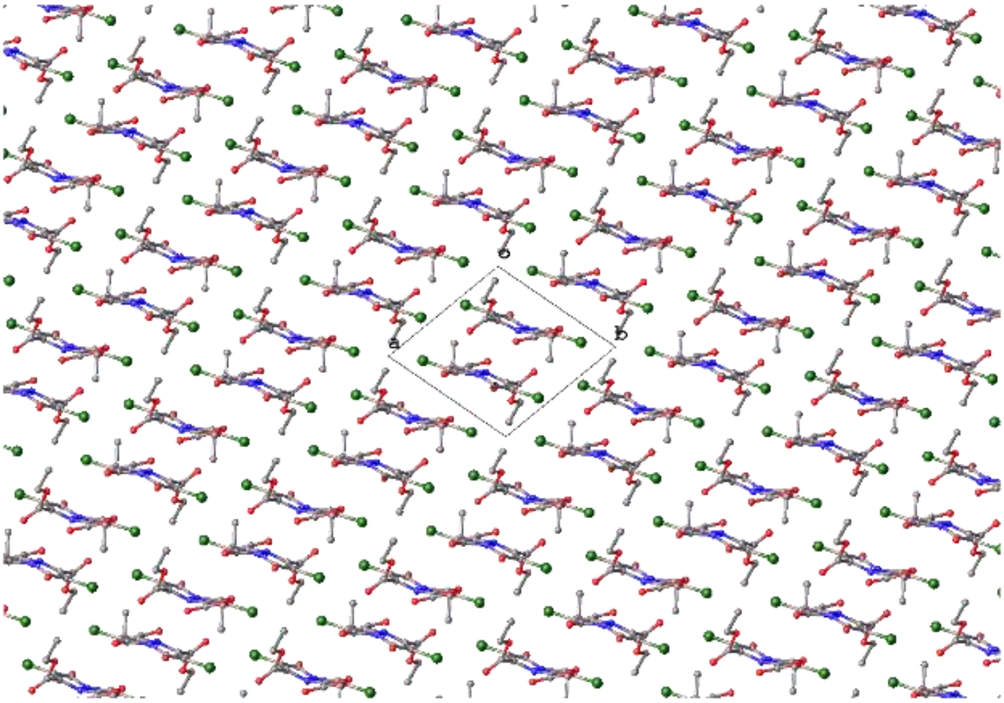

(a)

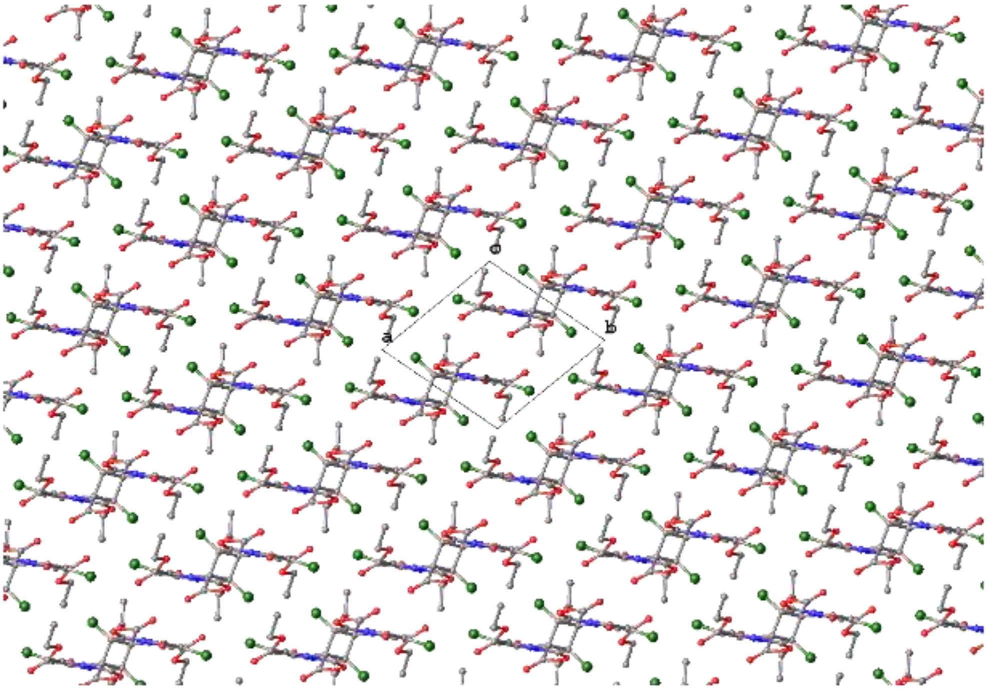

(b)

Figure S18. Schematic packing diagram of each moiety in **Cl_2B (C)** viewed normal to the *c*-axis, with (a) majority occupied bimane and (b) cycloaddition product. Hydrogen atoms omitted for clarity.

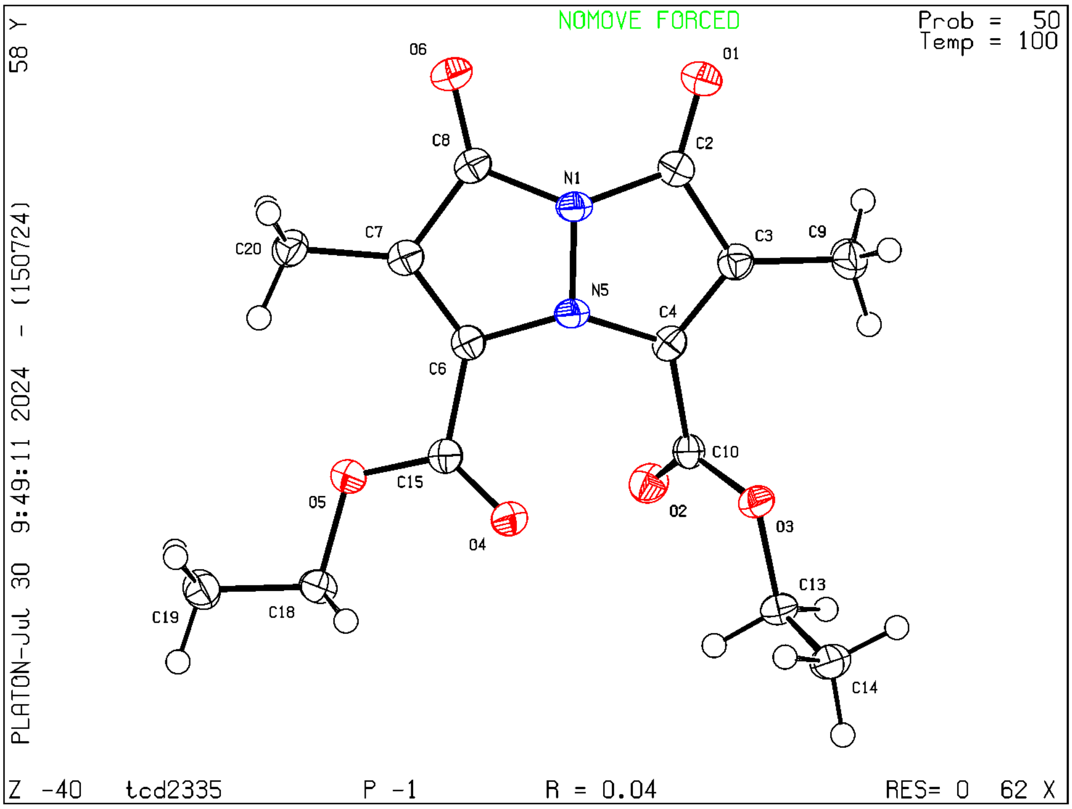

Figure S19. ORTEP plot of **Me_2B** with atoms labelled.

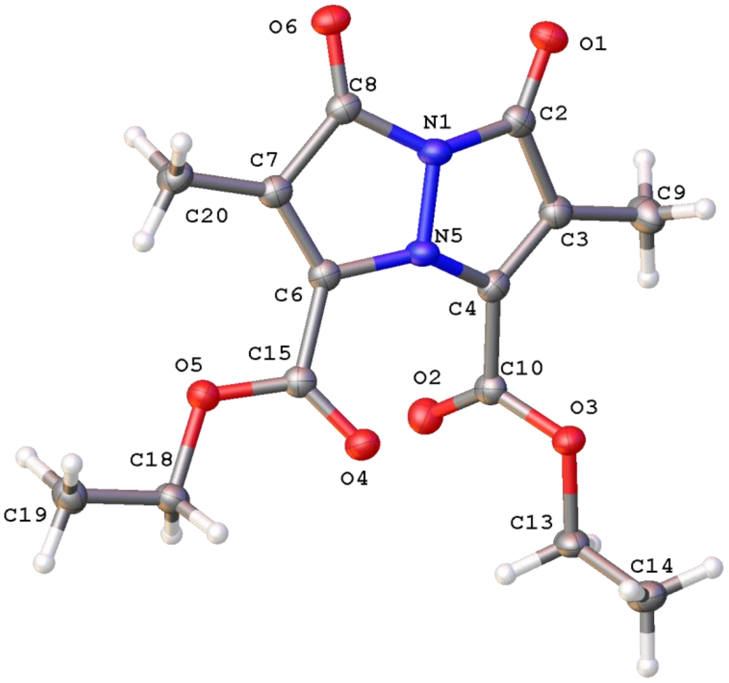

Figure S20. Molecular structure of **Me_2B** in the crystal. Displacement parameters shown at 50% probability.

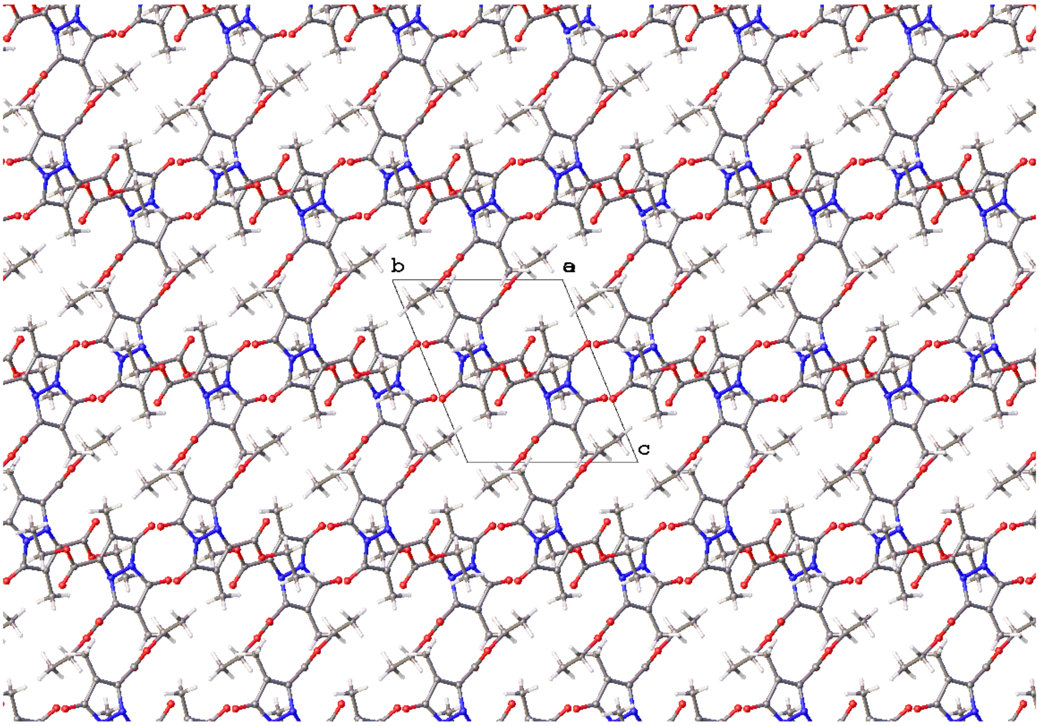

Figure S21. Schematic packing diagram of **Me2B**, viewed normal to the *a*-axis.

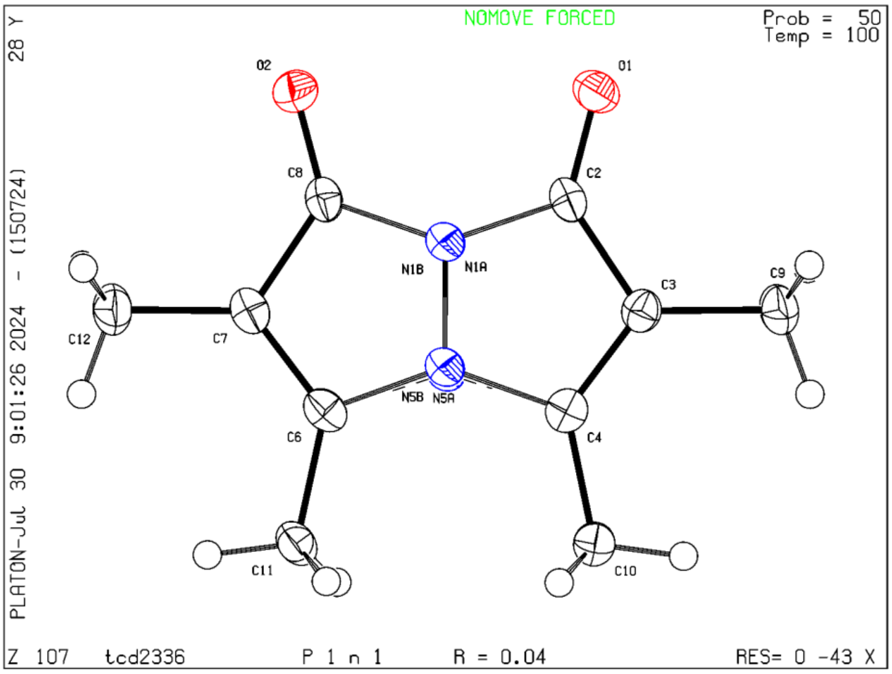

Figure S22. ORTEP plot of **Me4B** with atoms labelled.

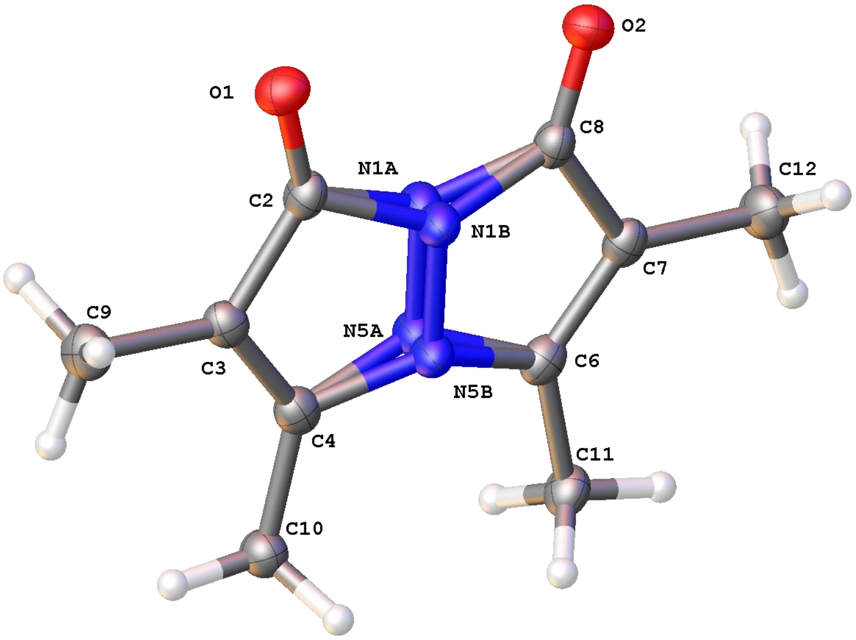

Figure S23. View of the molecular structure of **Me4B** in the crystal with disorder in the N–N bridge (54:46%). Displacement parameters shown at 50% probability.

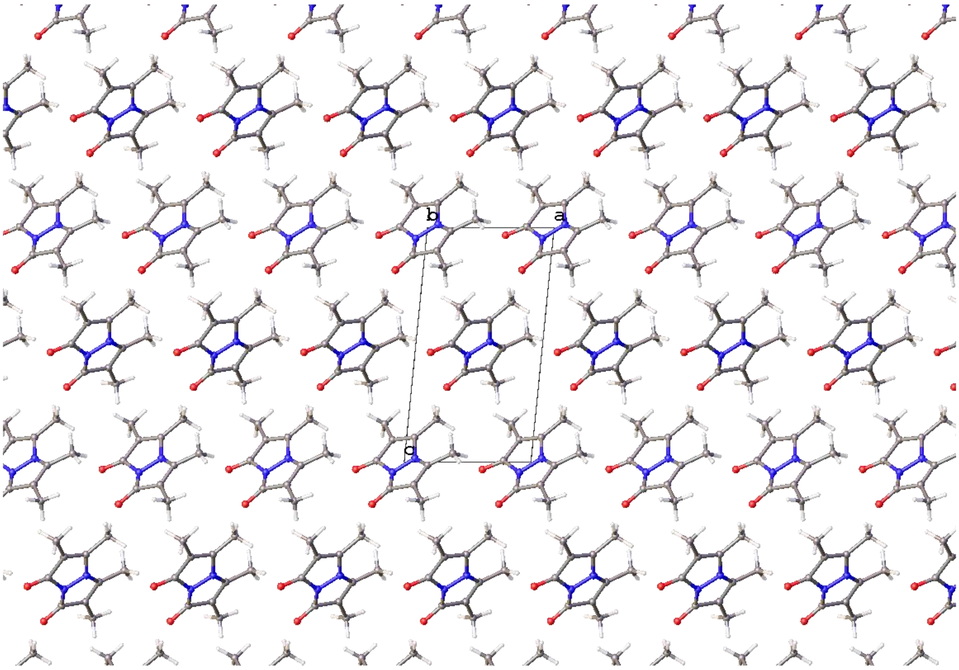

Figure S24. Schematic packing diagram of majority occupied moiety in **Me4B**, viewed normal to the *b*-axis.

Refinement details:

Me_4B; Similar to published structures (see CSD refcode TMZBCO10,[6] TMZBCO11[7]). In **Me_4B** there is disorder in the N-N position, refined with geometric restraints (SADI, RIGU) in two positions with 50% occupancy. Syn conformation.

Cl_2B (A); Whole molecule disorder with two different products, original bimane and cycloaddition product. Geometric restraints (RIGU, SADI) and displacement restraints (SIMU) and constraints (EADP, Cl's, C9, C12, C13, C17, O2, O11, O16) used in the model. Refined bimane occupancy, 58% and cycloaddition product 21%.

RIGU applied to all atoms. EADP applied to atom pairs in brackets: (Cl1A Cl1B) (Cl2A Cl2B) (C17A C17B) (C13A C13B) (C9A C9B) (C2A C2B) (O2A O2B) (O11A O11B) (O16A O16B)
SIMU applied to the following: N1A N1B C2A C2B C3A C3B C4A C4B N5A N5B C6A C6B C7A C7B C8A C8B.
SADI applied to the following 4 atoms in brackets: (C13B C12B C13A C12A) (C18A C17A C18B C17B) (O16B C17B O16A C17A) (O16B C14B O16A C14A) (O11B C12B O11A C12A).

Cl_2B (B); Molecule is 90% bimane, and 5% cycloaddition. Modelled in two locations using restraints (SADI, RIGU, SIMU) and constraints (EADP).

RIGU applied to the following: Cl1A O1A N5A C2A C4A C3A.
SIMU applied to the following: Cl1A O1A N5A C2A C4A C3A.
SADI applied to the following atoms in brackets: (C3B C2B C7A C8A), (N1B C2B N1A C8A), (C6A C7A C4B C3B), (Cl1B C3B Cl2A C7A), (O1B C2B O1B C8A) (C14B C6B C4A C9A), (N1B C8B N1A C2A), (C3A C2A C8B C7B), (N5B C6B N5A C4A), (O1A C8B O1A C2A) (O5B_$1 C17B_$1 O3A_$1 C17B_$1), (O6B C8B O1A C2A), (Cl1A C3A Cl2B C7B).
EADP applied to the following atoms in brackets: (C2A C8B), (C3A C7B), (C4A C6B), (O5B C6A C4B), (N5A N5B), (C14B C9A), (O4B O2A), (C4B C9B C6A C9B), (Cl2A Cl1B), (N1B N1A), (Cl1A Cl2B), (O1A O6B), (O1B O6A), (C2B C8A), (O1B O1B), (N5A N1A), (C7A C3B).

Cl_2B (C); Molecule is 63% bimane, and 18.5% cycloaddition. Modelled in two locations using restraints (SADI, RIGU, SIMU) and constraints (EADP).

RIGU applied to all atoms.
SADI applied to the following atoms in brackets: (O6B C12B O6A C12A), (SADI O6B C13 O6A C13), (SADI O5B C12B O5A C12A), (SADI O4A C9A O4B C9B), (SADI O3B C9B O3A C9A), (SADI C11A C10A C11B C10B), (SADI O1B C8B O2A C8A), (SADI C6A C12A C12B C6B).
ISOR applied to O2A O1B.
EADP applied to the following atoms in brackets: (O11A O11B), (EADP O16A O16B), (EADP O2A O1B), (EADP O6A O6B), (EADP C10B C10A), (EADP O4B O4A), (EADP C11B C11A), (EADP C12B C12A), (EADP O5B O5A), (EADP C7B C7A).
EXYZ applied to the following atoms in brackets: (O6A O6B), (O2A O1B).

Me4B; Disorder in the N-N bridge, modelled with geometrical restraints (SADI) and displacement constraints (EADP).

SADI applied to the following atoms in brackets: (N5B C6 N5A C6), (N1B C8 N1A C8), (N1B C2 N1A C2), (N5B C4 N5A C4).
EADP applied to the following: N1B N5B N1A N5A.

Crystallographic data for the structures reported here have been deposited with the Cambridge Crystallographic Data Centre. CCDC 2387334–2387338 contain the supplementary crystallographic data for this paper. These data can be obtained free of charge from The Cambridge Crystallographic Data Centre via www.ccdc.cam.ac.uk/structures.

Table S2: Details of XRD refinement.

Compound	Cl_2B (A)	Cl_2B (B)	Me_2B	Me_4B (B)	Cl_2B (C)29
**Local TCD number**	TCD2128	TCD2332	TCD2335	TCD2336	TCD2363
**CCDC number**	2387334	2387338	2387336	2387337	2387338
**Empirical formula**	$C_{12}H_{10}Cl_2N_2O_6$	$C_{12}H_{10}Cl_2N_2O_6$	$C_{14}H_{16}N_2O_6$	$C_{10}H_{12}N_2O_2$	$C_{24}H_{20}Cl_4N_4O_{12}$
**Formula weight**	349.12	349.12	308.29	192.22	698.24
**Temperature [K]**	100(2)	100(2)	100(2)	100(2)	100(2)
**Crystal system**	triclinic	triclinic	triclinic	monoclinic	triclinic
**Space group (number)**	$P\bar{1}$ (2)	$P\bar{1}$ (2)	$P\bar{1}$ (2)	Pn (7)	$P\bar{1}$ (2)
***a* [Å]**	8.5648(8)	8.6106(6)	8.6657(2)	6.5159(2)	8.5840(9)
***b* [Å]**	8.6208(6)	8.7061(6)	9.0821(2)	5.97180(10)	8.6260(8)
***c* [Å]**	9.8520(8)	9.8278(6)	10.9417(3)	12.1005(3)	9.8506(9)
**α [°]**	95.075(5)	95.569(3)	105.7848(14)	90	95.060(6)
**β [°]**	95.688(6)	94.695(3)	110.4679(14)	95.6458(14)	95.684(7)
**γ [°]**	105.568(6)	105.770(3)	102.6954(14)	90	105.622(7)
**Volume [$Å^3$]**	692.25(10)	701.14(8)	727.46(3)	468.57(2)	693.96(12)
Z	2	2	2	2	1
**ρ_{calc} [$g.cm^{-3}$]**	1.675	1.654	1.407	1.362	1.671
**μ [mm^{-1}]**	4.546	4.488	0.946	0.794	4.535
***F*(000)**	356	356	324	204	356
**Crystal size [mm^3]**	0.072×0.112×0.117	0.395×0.136×0.109	0.216×0.191×0.034	0.167×0.134×0.087	0.096×0.093×0.052
**Crystal colour**	clear yellow	yellow	yellow	yellow	yellow
**Crystal shape**	fragment	block	plate	prism	block
**Radiation**	Cu K_α (λ=1.54178 Å)	Cu K_α (λ=1.54178 Å)	Cu K_α (λ=1.54178 Å)	Cu K_α (λ=1.54178 Å)	Cu K_α (λ=1.54178 Å)
**2Θ range [°]**	9.09 to 140.66 (0.82 Å)	9.10 to 139.76 (0.82 Å)	9.33 to 139.55 (0.82 Å)	14.71 to 139.84 (0.82 Å)	9.09 to 141.85 (0.82 Å)
**Index ranges**	−10 ≤ h ≤ 10<br>−10 ≤ k ≤ 10<br>−11 ≤ l ≤ 12	−10 ≤ h ≤ 10<br>−9 ≤ k ≤ 10<br>−11 ≤ l ≤ 11	−10 ≤ h ≤ 10<br>−11 ≤ k ≤ 10<br>−12 ≤ l ≤ 13	−7 ≤ h ≤ 7<br>−7 ≤ k ≤ 7<br>−14 ≤ l ≤ 14	−10 ≤ h ≤ 8<br>−9 ≤ k ≤ 10<br>−11 ≤ l ≤ 11
**Reflections collected**	8665	8622	8937	6450	9876
**Independent reflections**	2576<br>R_{int} = 0.0519<br>R_{sigma} = 0.0497	2617<br>R_{int} = 0.0447<br>R_{sigma} = 0.0423	2722<br>R_{int} = 0.0334<br>R_{sigma} = 0.0307	1530<br>R_{int} = 0.0329<br>R_{sigma} = 0.0300	2607<br>R_{int} = 0.0525<br>R_{sigma} = 0.0468
**Completeness**	99.1 %	99.4 %	99.7 %	100.0 %	99.4 %
**Data / Restraints / Parameters**	2576 / 435 / 348	2617/69/241	2722/0/203	1530/6/133	2607/334/287
**Absorption correction T_{min}/T_{max} (method)**	0.6117 / 0.7533<br>(multi-scan)	0.5111/0.7533<br>multi-scan	0.6581/0.7533<br>(multi-scan)	0.6936/0.7533<br>(multi-scan)	0.6305/0.7533<br>(multi-scan)
**Goodness-of-fit on F^2**	1.064	1.041	1.062	1.096	1.036
**Final *R* indexes [I≥2σ(I)]**	R_1 = 0.0556<br>wR_2 = 0.1541	R_1 = 0.0496<br>wR_2 = 0.1330	R_1 = 0.0379<br>wR_2 = 0.1034	R_1 = 0.0360<br>wR_2 = 0.0966	R_1 = 0.0590<br>wR_2 = 0.1555
**Final *R* indexes [all data]**	R_1 = 0.0630<br>wR_2 = 0.1611	R_1 = 0.0513<br>wR_2 = 0.1346	R_1 = 0.0407<br>wR_2 = 0.1064	R_1 = 0.0367<br>wR_2 = 0.0973	R_1 = 0.0722<br>wR_2 = 0.1670
**Largest peak/hole [$eÅ^{-3}$]**	0.57/−0.39	0.71/−0.34	0.30/−0.23	0.22/−0.14	0.44/−0.33
**Flack X parameter**				−0.2(3)	

6. References

1. Kosower, E. M.; Faust, D.; Ben-Shoshan, M.; Goldberg, I. *J. Org. Chem.* **1982**, *47*, 214–221.
2. Kosower, E. M.; Pazhenchevsky B. *J. Am. Chem. Soc.* **1980**, *102*, 4983–4993.
3. Neogi, I.; Grynszpan, F.; Das, P. J. *Synlett* **2018**, *29*, 1043–1046.
